# Visualizing translation dynamics at atomic detail inside a bacterial cell

**DOI:** 10.1038/s41586-022-05255-2

**Published:** 2022-09-28

**Authors:** Liang Xue, Swantje Lenz, Maria Zimmermann-Kogadeeva, Dimitry Tegunov, Patrick Cramer, Peer Bork, Juri Rappsilber, Julia Mahamid

**Affiliations:** 1 Structural and Computational Biology Unit, European Molecular Biology Laboratory (EMBL), Heidelberg, Germany; 2 Collaboration for joint PhD degree between EMBL and Heidelberg University, Faculty of Biosciences, Heidelberg, Germany; 3 Chair of Bioanalytics, Technische Universität Berlin, Berlin, Germany; 4 Department of Molecular Biology, Max-Planck-Institute for Biophysical Chemistry, Göttingen, Germany; 5 Yonsei Frontier Lab, Yonsei University, Seoul, South Korea; 6 Department of Bioinformatics, Biocenter, University of Würzburg, Würzburg, Germany; 7 Wellcome Centre for Cell Biology, University of Edinburgh, Edinburgh, UK

**Keywords:** Cryoelectron tomography, Bacterial structural biology, Cryoelectron tomography, Ribosome

## Abstract

Translation is the fundamental process of protein synthesis and is catalysed by the ribosome in all living cells^1^. Here we use advances in cryo-electron tomography and sub-tomogram analysis^2,3^ to visualize the structural dynamics of translation inside the bacterium *Mycoplasma pneumoniae*. To interpret the functional states in detail, we first obtain a high-resolution in-cell average map of all translating ribosomes and build an atomic model for the *M.* *pneumoniae* ribosome that reveals distinct extensions of ribosomal proteins. Classification then resolves 13 ribosome states that differ in their conformation and composition. These recapitulate major states that were previously resolved in vitro, and reflect intermediates during active translation. On the basis of these states, we animate translation elongation inside native cells and show how antibiotics reshape the cellular translation landscapes. During translation elongation, ribosomes often assemble in defined three-dimensional arrangements to form polysomes^4^. By mapping the intracellular organization of translating ribosomes, we show that their association into polysomes involves a local coordination mechanism that is mediated by the ribosomal protein L9. We propose that an extended conformation of L9 within polysomes mitigates collisions to facilitate translation fidelity. Our work thus demonstrates the feasibility of visualizing molecular processes at atomic detail inside cells.

## Abstract (web summary)

Cryo-electron tomography is used to reveal the structural dynamics and functional diversity of translating ribosomes in *Mycoplasma pneumoniae*, providing insight into the translation elongation cycle inside cells and how it is reshaped by antibiotics.

## Main

Translation of genetic information through messenger RNAs (mRNAs) into proteins is performed by the ribosome, one of the primordial macromolecular machines in cells^1^. The ribosome consists of a small and a large subunit (30S and 50S in prokaryotes), which form the aminoacyl (A), peptidyl (P) and exit (E) transfer RNA (tRNA) binding sites at their interface. The translation process can be divided into four phases: initiation, elongation, termination and recycling^1,5^. During the elongation phase, the ribosome undergoes a fundamental cycle to add one amino acid to the nascent peptide chain, which can be subdivided into three steps: decoding, peptidyl transfer and translocation. These steps involve structural changes in the ribosome that include subunit rotations, elongation factor association and tRNA accommodation^1,5^. Many intermediates during the elongation cycle have been identified on the basis of structures derived by cryo-electron microscopy (cryo-EM) and computational image classification^6–16^. Most available structures, however, are of ribosomes that were isolated from model bacteria such as *Escherichia coli* and *Thermus thermophilus*, and were often trapped in specific states by antibiotics, GTP analogues or mutations^1,5,17^. Thus, a detailed structural description of the translation process within the native cellular context is lacking. Although actively translating ribosomes have been visualized inside cells by cryo-electron tomography (cryo-ET), the maps generated in previous studies were limited to resolutions on the nanometre scale^2,18–22^. We have recently developed image-processing algorithms for cryo-ET that make it possible to resolve stalled ribosomes to residue level inside the genome-reduced bacterium *M.* *pneumoniae*^3^. Here, we use these technical advances to perform large-scale structure classification and spatial analysis of ribosomes, to visualize parts of the translation process in great detail inside *M.* *pneumoniae* as a prokaryotic minimal cell model^18^.

## In-cell structure of the *M. pneumoniae* ribosome

**Fig. 1:**
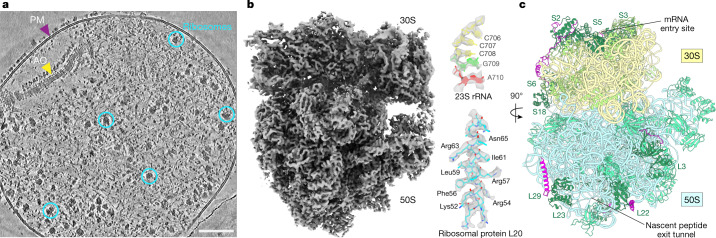
Ribosome structure in *M.* *pneumoniae* cells. **a**, A denoised tomographic slice of a *M.* *pneumoniae* cell. AO, attachment organelle; PM, plasma membrane. Examples of ribosomes are circled. The representative tomogram (selected from 15) was acquired with a Volta phase plate for better visualization of the cellular morphology. Similar imaging conditions, excluding the use of a phase plate, were used for the acquisition of all tomograms for subsequent analysis. Scale bar, 100 nm. **b**, A 3.5-Å in-cell ribosome map (left) shows well-resolved rRNA bases and ribosomal protein side chains (right). **c**, An atomic model of the *M.* *pneumoniae* ribosome shows structural similarity to other bacterial ribosomes. Eleven ribosomal proteins (dark green) have sequence extensions (magenta).

**Extended Data Fig. 1:**
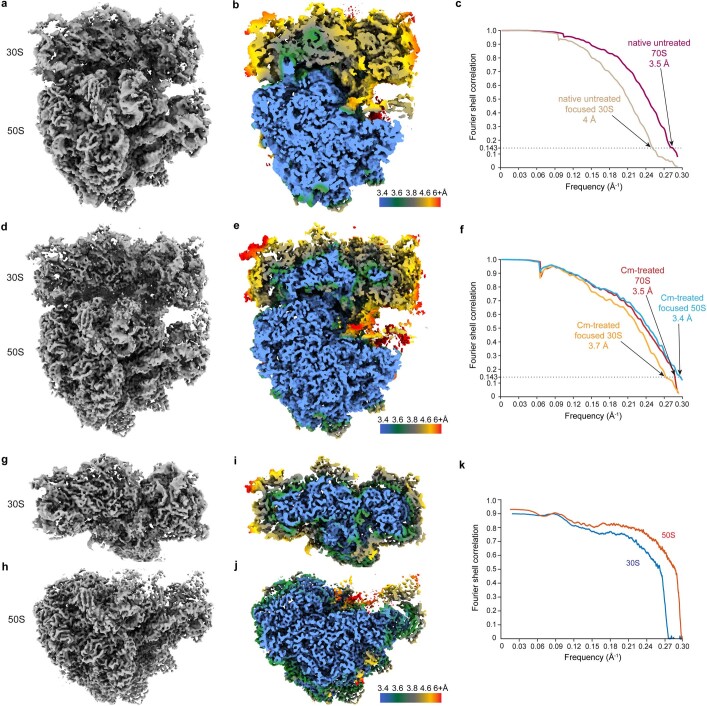
*M. pneumoniae* in-cell ribosome maps. **a**, 70S ribosome map determined from 77,539 sub-tomograms from 356 untreated *M. pneumoniae* cells. **b**, Map coloured by local resolutions. 50S has the highest local resolutions as it dominates the overall alignment. The relatively lower local resolutions of the 30S indicate their high flexibility during active translation. **c**, Fourier shell correlation (FSC) curves for global 70S and focused 30S refinement, and the reported resolution value at FSC = 0.143. The Nyquist limit for the data is 3.4 Å. **d**, 70S ribosome map determined from 18,987 sub-tomograms from 65 Cm-treated cells. **e**, Map coloured by local resolutions. **f**, FSC curves of the Cm-treated 70S ribosome, and of focused refinements on 30S and 50S respectively. **g**-**h**, Focused refined 30S and 50S maps from the Cm-treated dataset. **i**-**j**, The corresponding local-resolution maps. Atomic models for 30S and 50S were first built based on maps in **g** and **h**. **k**, Model-to-map FSC curves for 30S and 50S, respectively.

**Extended Data Fig. 2:**
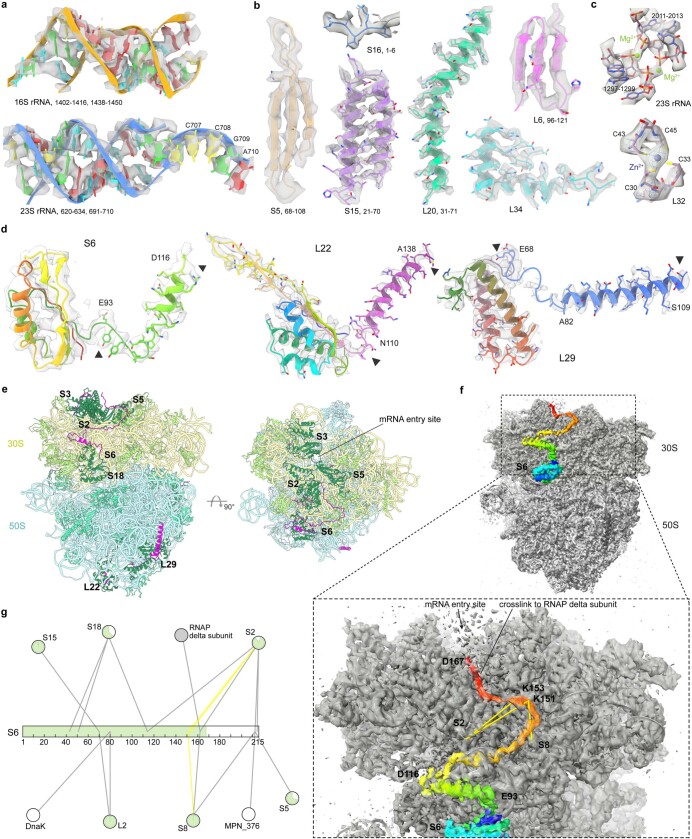
Structural features of the *M. pneumoniae* ribosome. **a**,**b**, Examples of regions of the atomic model fitted into the density map for rRNAs (**a**) and proteins (**b**). **c**, Densities corresponding to ions are also observed. **d**, Extensions of ribosomal proteins S6, L22 and L29 form secondary structures (between arrowheads) that were clearly resolved in the map. **e**, The 70S model showing ribosomal proteins with sequence extensions (dark green). The extensions built in the model are highlighted in magenta. In addition to the helix-forming sequences of S6, L22 and L29, some loops can be traced, especially the long C-terminal loop of S6. **f**, Whole-cell cross-linking mass spectrometry data confirms the model built for the long loop extension of S6. **g**, The cross-linking network of S6 identified in the previous work^2^, with the sequence range (1–167) resolved in the map coloured in green.

**Extended Data Fig. 3:**
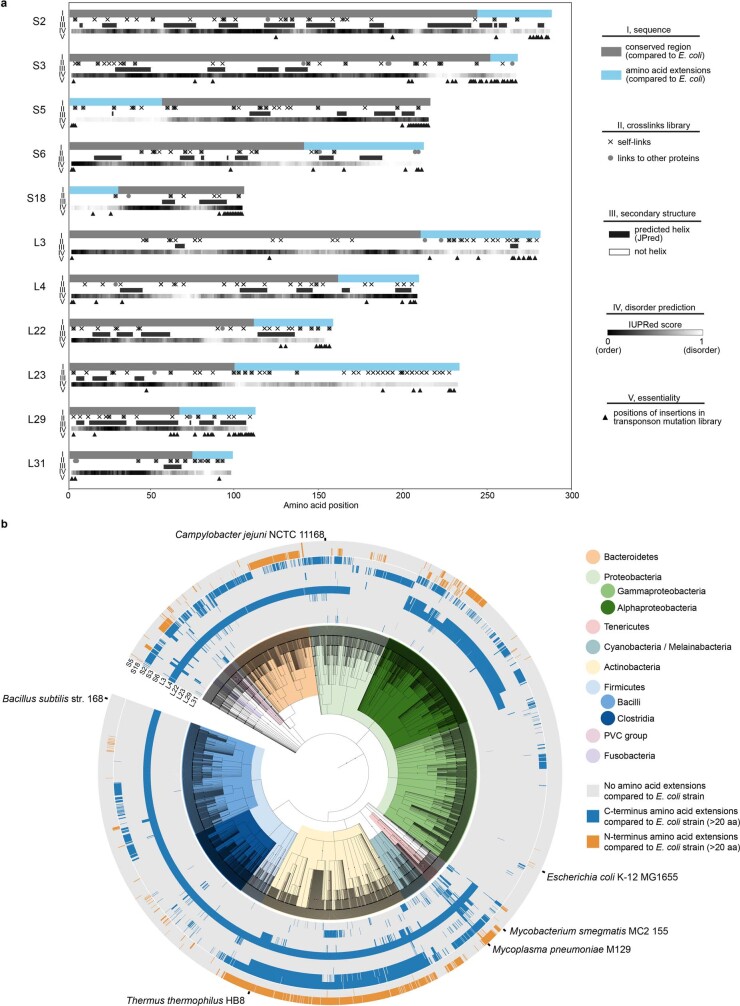
Ribosomal protein extensions. **a**, Eleven ribosomal proteins in *M. pneumoniae* have sequence extensions compared to *E. coli*. Along the sequences, cross-links identified by in-cell cross-linking mass spectrometry, secondary structure prediction, disorder prediction, and sequence essentiality prediction based on a transposon mutation library are displayed (quantified in Supplementary Table 2). **b**, Extensions in the eleven ribosomal proteins are found throughout bacteria, but are not specific to any sub-groups.

To investigate the structural details of the translation machinery, we first obtained 3.5-Å consensus maps by averaging all ribosomes detected in cryo-electron tomograms of intact *M.* *pneumoniae* cells (Fig. 1a,b, Extended Data Fig. 1, Supplementary Table 1, Supplementary Discussion and Methods). Focused refinements on the 30S and 50S subunits improved the map quality and revealed well-resolved ribosomal RNA (rRNA) bases and ribosomal protein amino acid side chains (Fig. 1b and Extended Data Fig. 2a–c). The high-resolution in-cell consensus maps allowed us to build de novo an atomic model for the *M.* *pneumoniae* ribosome (Extended Data Fig. 2d–g and Methods). The structure shows high similarity to other bacterial ribosomes, but also reveals several new features (Fig. 1c and Extended Data Fig. 2d–g). Specifically, 11 of the 52 ribosomal proteins in *M.* *pneumoniae* have extended sequences compared to *E.* *coli* (Extended Data Fig. 3a and Supplementary Discussion). Most of these extensions are predicted to be disordered, but those of ribosomal proteins S6, L22 and L29 form secondary structures and were built in the model (Fig. 1c and Extended Data Figs. 2d–e and 3a). We found such extensions to be common throughout the bacterial kingdom (Extended Data Fig. 3b, Supplementary Discussion and Supplementary Table 2), possibly representing ribosome diversity in adaptation to different environments and lifestyles^23^. Although the functions of these extensions remain largely elusive, their disruption affects cellular fitness or survival in *M.*  *pneumoniae*^24^ (Supplementary Discussion). Thus, the high-resolution ribosome map and the atomic model derived from intact *M.* *pneumoniae* provide the basis to investigate in detail the conformational and compositional changes of ribosomes during translation inside cells.

## Structural dynamics of translation in cells

**Fig. 2:**
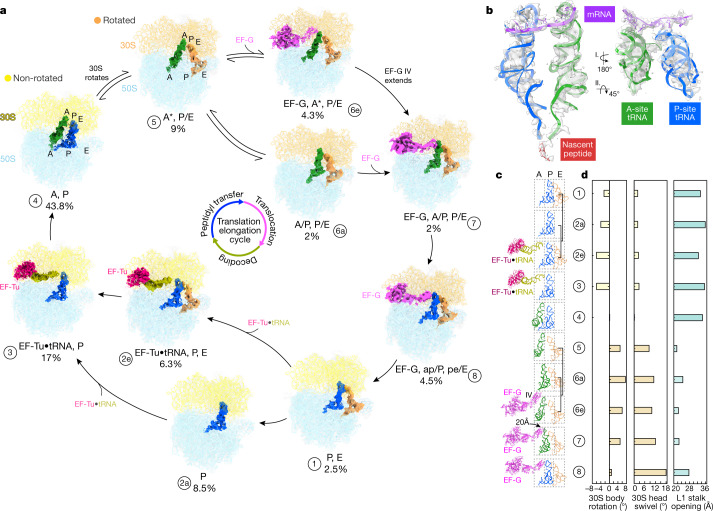
Ribosome classification reconstructs the translation elongation cycle. **a**, Ten 70S structures determined inside *M.* *pneumoniae* cells represent translation elongation intermediate states, which are characterized by the binding of different elongation factors (EFs) and tRNAs. The frequencies of occurrence for different intermediates are calculated from the classified particle numbers (Extended Data Fig. 4). **b**, Densities for mRNA, tRNAs and the nascent peptide chain are well-resolved in the most-populated 'A, P' state. **c**, Trajectories of the elongation factors EF-Tu and EF-G and the A-, P- and E-site tRNAs along translation elongation. **d**, Major conformational changes of the ribosome along the elongation trajectory: 30S body rotation, 30S head swivel and L1 stalk opening.

**Extended Data Fig. 4:**
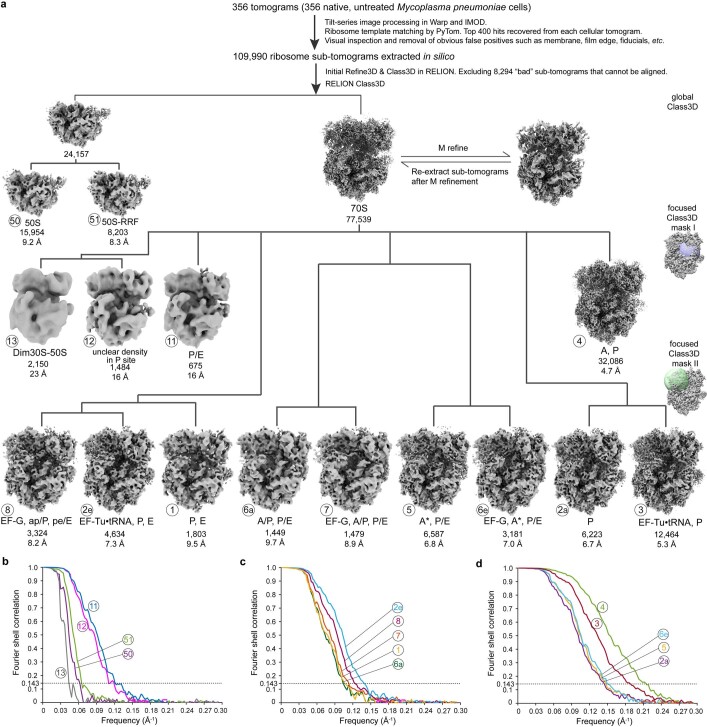
Classification and refinement of ribosomes in native untreated cells. **a**, A diagram of the image-processing workflow. The classification process can be grouped into three tiers: global classification, focused classification on the tRNA path region (mask I), and focused classification on elongation factor and A/T tRNA binding region (mask II). For each class, a unique number identifier and class name are assigned for tracking. The particle number and the global map resolution at FSC = 0.143 are provided for each class. **b**-**d**, FSC curves for all refined maps.

**Extended Data Fig. 5:**
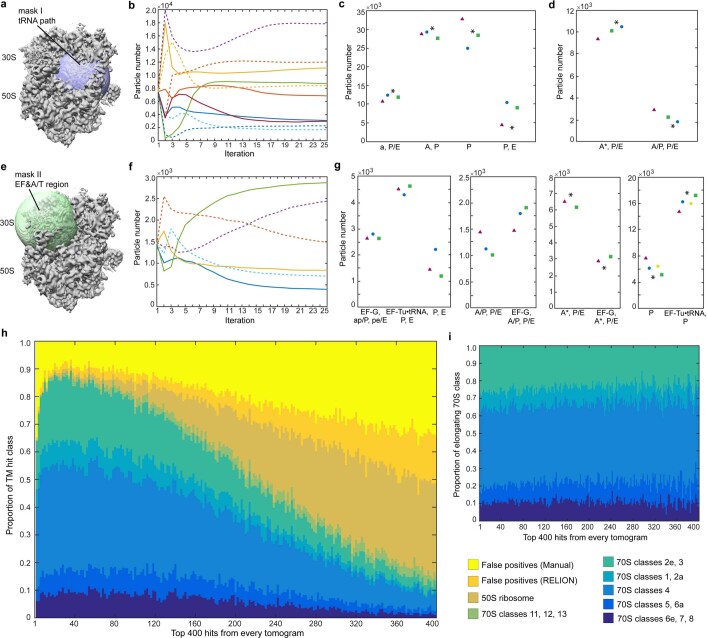
Validation of ribosome detection and classification. **a**, Mask I for focused classification on tRNA path region. **b**, A representative RELION classification job with mask I. Each line indicates the change in particle numbers in one class over 25 iterations. Classes that show the same structure were grouped according to the tRNA occupancy ('a, P/E', 'P, E', 'P', 'A, P'). 'a, P/E' contains heterogeneous density around the A site. **c**, Results of the classification job as shown in **b**, and of three additional parallel jobs. **d**, Results of following classification jobs that further classify the 'a, P/E' class into 'A*, P/E' and 'A/P, P/E'. **e**, Mask II for focused classification on elongation factor (EF) and A/T tRNA sites. **f**, Changes in particle numbers over iterations in a representative classification job with the mask II. **g**, Results of parallel focused classification jobs with mask II. **h**, Distribution of the ribosome classes against template matching cross-correlation scores used for ribosome localization. For each of the 356 tomograms of untreated cells, the 400 highest scoring hits were extracted and ranked. Obvious false positives were manually excluded first. Additional false positives were identified during RELION classification. 70S classes that are structurally similar were grouped in the plot for better visualization. **i**, Same as **h**, but only showing the 70S classes in the elongation phase. The proportions of different 70S classes remain stable across the top 400 hits, demonstrating that the classification results are not biased by the ribosome picking.

**Extended Data Fig. 6:**
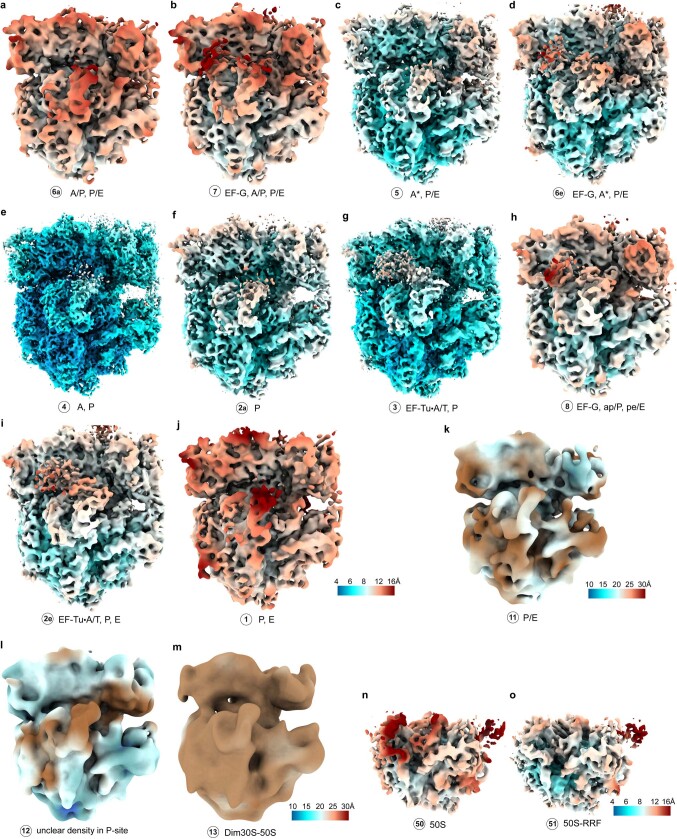
Local-resolution maps for ribosome classes in untreated cells. Maps of the 15 classes determined in the dataset of untreated cells (Extended Data Fig. 4), coloured by local resolution calculated in RELION.

**Extended Data Fig. 7:**
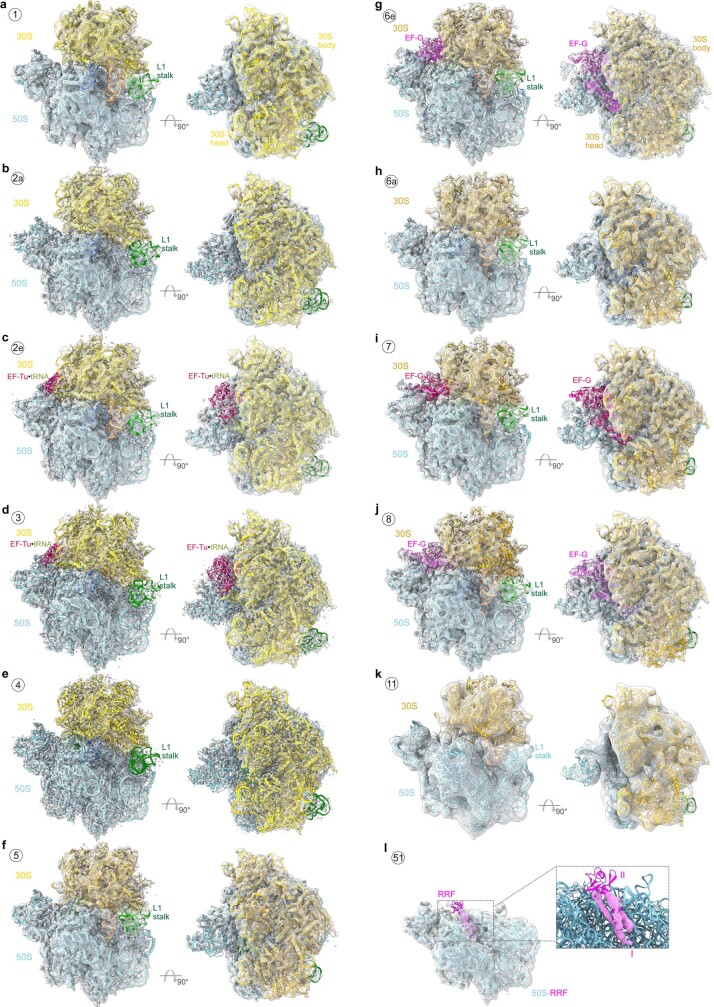
Model building for ribosome classes in untreated cells. **a**-**j**, Models of the ten ribosome classes in the elongation phase constructed by flexible fitting. **k**, The model for the ribosome with hybrid P/E-site tRNA. **l**, Free 50S in complex with ribosome recycling factor (RRF).

To analyse the structural changes that are associated with the translation process inside cells, we performed computational classification of 101,696 ribosomes from 356 tomograms of native cells, and obtained 13 distinct ribosome classes (Extended Data Fig. 4, Supplementary Table 3 and Methods). Ten classes determined at resolutions ranging from 4 to 10 Å were assigned to the translation elongation phase on the basis of elongation factor and tRNAs binding to the ribosome (Fig. 2a and Extended Data Figs. 4–7). The remaining three classes represent 70S with a single P/E-site tRNA, 50S in complex with the ribosome recycling factor, and free 50S subunits (Extended Data Figs. 4–7 and Supplementary Discussion). The ten classes within the elongation phase account for 70% of the detected ribosomes, consistent with the expectation that most ribosomes inside living cells are engaged in the elongation phase, which lasts considerably longer than the initiation, termination and recycling phases^5,25^.

**Extended Data Fig. 8:**
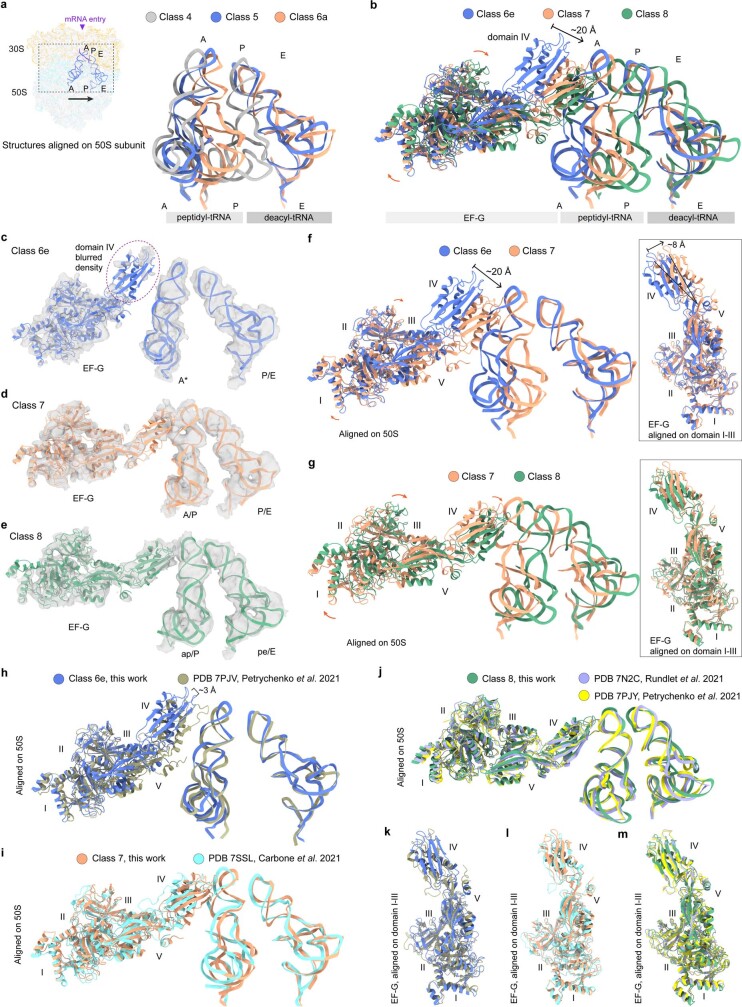
Early-to-late translocation intermediates during elongation. **a**, Early translocation intermediates prior to EF-G binding. Only tRNAs in the aligned class models are displayed. **b**, Early-to-late translocation intermediates in the presence of EF-G. Continuous structural changes are observed from class 6e to 7 to 8, including a roughly 20 Å movement of EF-G’s domain IV toward the A site (black arrow), an overall rotation of the entire EF-G (orange arrows) and inter-domain conformational changes. **c**-**e**, Densities and models for EF-G and tRNAs in classes 6e, 7 and 8. In class 6e, EF-G’s domain IV is less resolved in the map (red circle). It may contain a mixture of intermediate states with domain IV moving toward the A site. The fitted model represents the average position. Further classification could not address the high flexibility. **f**, From class 6e to 7, EF-G undergoes a small overall rotation (orange arrows) and an inter-domain rearrangement. Inset (rotated view, aligned on EF-G domains I-III) shows rotation of domain IV relative to other domains. Movement of EF-G’s domain IV towards the A site results from both overall rotation on the ribosome and inter-domain rearrangement. **g**, From class 7 to 8, EF-G rotates as one body without significant inter-domain rearrangement (inset, rotated view, aligned on EF-G domains I-III). **h**, Class 6e shows structural similarity to a reported early translocation state 'H1-EF-G–GDP-pi' (PDB 7PJV) determined by Petrychenko *et al*. 2021^15^. **i**, Class 7 resembles 'PRE–EF-G–GDP–Pi' (PDB 7SSL) reported by Carbone *et al*. 2021^16^, and 'INT1' (PDB 7N2V, not shown) by Rundlet *et al*. 2021^14^. **j**, Class 8 resembles the late translocation intermediates reported by Rundlet *et al*. 2021^14^ ('INT2', PDB 7N2C) and Petrychenko *et al*. 2021^15^ ('CHI1-EF-G-GDP', PDB 7PJY). **k**-**m**, Minor inter-domain conformational difference found between EF-Gs in our models and those in the reported in vitro structures.

The identified elongation classes can be ordered to reconstruct the translation elongation cycle^1,5^. By flexible fitting of our *M.* *pneumoniae* ribosome atomic model into the classified maps (Methods and Supplementary Table 3), we obtained pseudo-atomic models that delineate conformational and compositional changes of the ribosome complex, including rotations of 30S body and head, coordination of elongation factors, movement of tRNAs through the A–P–E sites and dynamics of the L1 stalk (Fig. 2, Extended Data Fig. 7a–j and Supplementary Videos 1 and 2). The number of ribosomes within each of the classes provides the relative abundance of translation elongation intermediates, which together reflect a steady state distribution determined by their relative rates of formation and depletion inside the cells (Fig. 2a and Extended Data Fig. 4a). The classic, non-rotated 'A, P' state was the most populated and determined at the highest resolution, showing clear density for mRNA, tRNAs and the nascent peptide (Fig. 2b). In the following states, we observed the oscillation of A- and P-site tRNAs into the hybrid A/P and P/E state, coupled with 30S subunit rotations and L1 stalk movement (Fig. 2a,c,d and Extended Data Fig. 7a–j), consistent with in vitro studies^6,8,9,12,26–30^. Along this trajectory, we also determined a partial hybrid 'A*, P/E' state with rotated 30S and deacyl-tRNA in the hybrid P/E site, but with only marginally relocated peptidyl-tRNA in the A site (Fig. 2a,d and Extended Data Fig. 8a), similar to a previously reported pre-translocational H2* state^8,14,26^. When viewed in the context of this trajectory, the high enrichment of the 'A, P' state could suggest that inter-subunit rotation represents a rate-limiting step for translation elongation in *M.* *pneumoniae*. However, in view of the entirety of the elongation cycle, processive inter-subunit rotation leading to efficient translocation requires the binding of the elongation factor EF-G. Notably, EF-G was found to bind to the ribosome in either the partial hybrid or the full hybrid states (Extended Data Fig. 8b–m). EF-G in the partial hybrid state (class 6e) is less extended and its domain IV does not overlap with the A site compared to the full hybrid state (Extended Data Fig. 8b–h). This EF-G-bound partial hybrid state therefore resembles early translocation intermediates before phosphate release^9,14–16^. In the following full hybrid state (class 7), domain IV of EF-G extends about 20 Å towards the A site, owing to both the rotation of entire EF-G and its inter-domain rearrangement (Fig. 2c and Extended Data Fig. 8f). Class 8 next shows a reverse rotation of the 30S body, the largest magnitude for 30S head swivel, the fully extended EF-G and the chimeric 'ap/P, pe/E' tRNAs (Fig. 2c,d and Extended Data Fig. 8g, j), indicating that it is a late translocation intermediate^9,10,14–16,28^. We thus demonstrate the existence of continual intermediates from early to late translocation stages during active translation elongation inside cells. Two EF-Tu-associated structures were also determined, with and without E-site tRNA (Fig. 2a and Extended Data Fig. 7c,d). This suggests that the binding of EF-Tu•tRNA to the ribosome and the disassociation of E-site tRNA are independent of each other. The 'P, E' class has a relatively low abundance, which suggests that the E-site tRNA is not stable and tends to disassociate quickly after translocation. This is in agreement with previous single-molecule fluorescence studies showing rapid release of the E-site tRNA^31,32^, and helps to resolve a long-standing controversy over its disassociation time point^1,12,33^. In summary, these results recapitulate major steps in translation elongation that have been defined by controlled in vitro studies and reconstruct the structural dynamics of the elongation cycles inside cells, highlighting the possible various paths of the reaction catalysed by the ribosome complex. Furthermore, with these results, a probability map for the occurrence of intermediate states can be derived to illustrate a putative energy landscape of translation elongation in cells. Energy landscapes of translation are suggested to be rugged^8,12,34,35^, depending on factors such as Mg^2+^ concentrations^36,37^, the presence of EF-G^34,38^ and temperature^8^. In *M.* *pneumoniae*, the intracellular concentrations of ribosomes, translation elongation factors and substrates can be up to ten times lower than those in *E.* *coli* (Supplementary Discussion). It is therefore possible that the energy landscapes of translation vary for different organisms, cell types and conditions. Applying the analysis introduced here to other cell types and conditions can deepen our understanding of the translation process within the native cellular context.

## Antibiotics alter translation landscapes

**Fig. 3:**
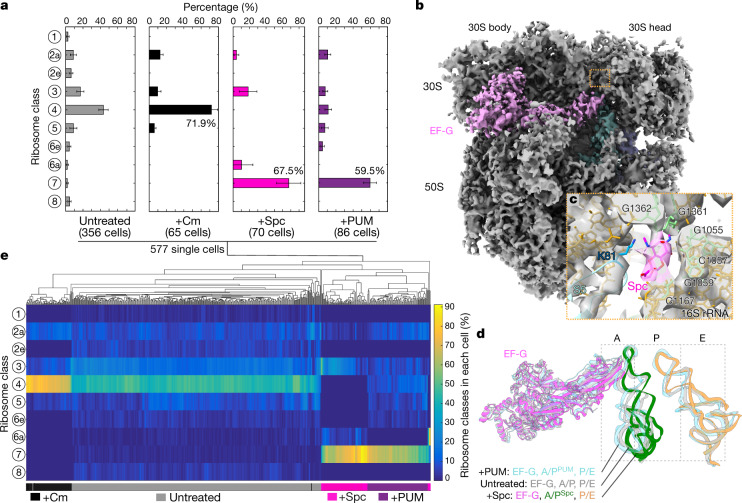
Antibiotics induce distinct translation elongation landscapes in cells. **a**, Distribution of translation elongation intermediates in native untreated cells, and in cells treated with three different antibiotics. Bar and whiskers indicate mean and s.d. for each class across all cells in the different treatment groups: untreated (*n* = 356 cells); +Cm, chloramphenicol-treated (*n* = 65 cells); +Spc, spectinomycin-treated (*n* = 70 cells); and +PUM, pseudouridimycin-treated (*n* = 86 cells). **b**, Ribosomes in Spc-treated cells are largely stalled in the 'EF-G, A/P^Spc^, P/E' state. **c**, The Spc molecule (magenta) is well-resolved and built in the 'EF-G, A/P^Spc^, P/E' ribosome model. It is surrounded by several 16S rRNA bases and loop 2 of ribosomal protein S5 near the 30S neck. **d**, The major state in Spc-treated cells is similar to the 'EF-G, A/P^PUM^, P/E' class in PUM-treated cells (light blue) and the 'EF-G, A/P, P/E' class in untreated cells (light grey), differing only in the position of the A/P-site tRNA on the 50S side. **e**, Single-cell clustering analysis on the basis of the translation elongation states of 577 individual cells under native and different antibiotic treatment conditions.

**Extended Data Fig. 9:**
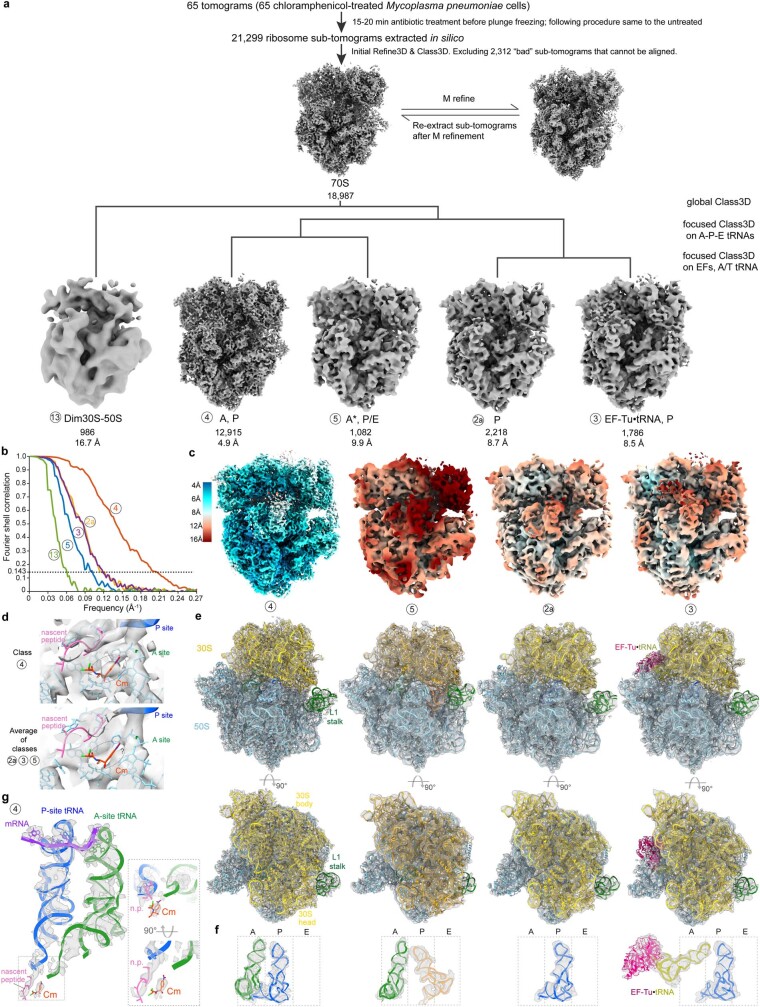
Classification, refinement and modelling of ribosomes in Cm-treated cells. **a**, Image-processing workflow for the Cm-treated dataset. The sub-tomogram classification and refinement are similar to those developed for the untreated dataset (Extended Data Fig. 4a). Heterogeneity may exist in the minor classes (2a, 3, 5), but the low particle numbers hindered further classification. **b**, FSC curves for all classes calculated following RELION refinements. **c**, Local-resolution maps. **d**, Density of the Cm molecule is resolved in the major 'A, P' class, but not in the other three minor classes owing to relatively low resolutions of these maps. **e**, Models built for the four classes, fitted into their corresponding densities. **f**, Elongation factors and tRNAs in the four classes. **g**, In the predominant 'A, P' class, mRNA, A- and P-site tRNA, the nascent peptide chain, and the Cm drug are well-resolved. The nascent chain has strong continuous density linked to the P-site tRNA, resulting from the inhibition of peptidyl transfer by the Cm molecule.

**Extended Data Fig. 10:**
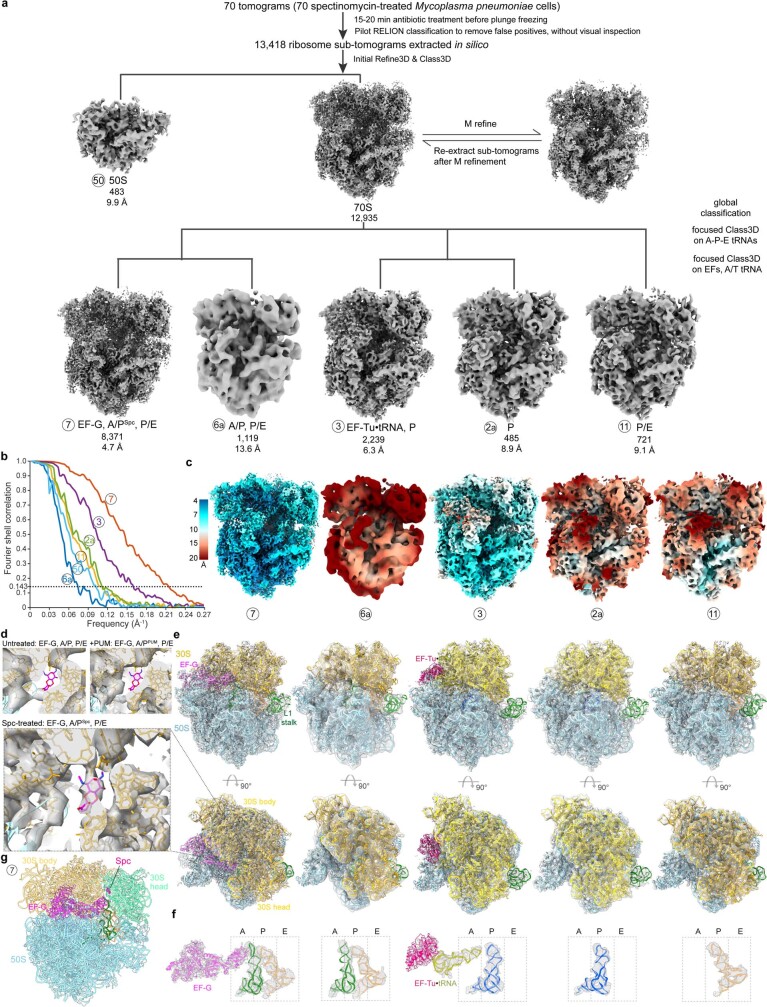
Classification, refinement and modelling of ribosomes in Spc-treated cells. **a**, Image-processing workflow for the Spc-treated dataset. The sub-tomogram classification and refinement are similar to those developed for the untreated dataset (Extended Data Fig. 4a), except for exclusion of the manual inspection step after template matching. **b**, FSC curves for all classes calculated following refinements. **c**, Local-resolution maps for Spc-treated 70S classes. **d**, Density corresponding to the Spc molecule (magenta) is clearly resolved in the major 'EF-G, A/P^Spc^, P/E' class. Densities from the untreated and PUM-treated data, where Spc is not present, are shown for comparison. **e**, Models built for the five Spc-treated 70S classes. **f**, Elongation factors and tRNAs in the five classes. **g**, The model of 'EF-G, A/P^Spc^, P/E' shows Spc binds to the 30S neck region, confirming its role in inhibiting 30S head dynamics and mRNA translocation.

**Extended Data Fig. 11:**
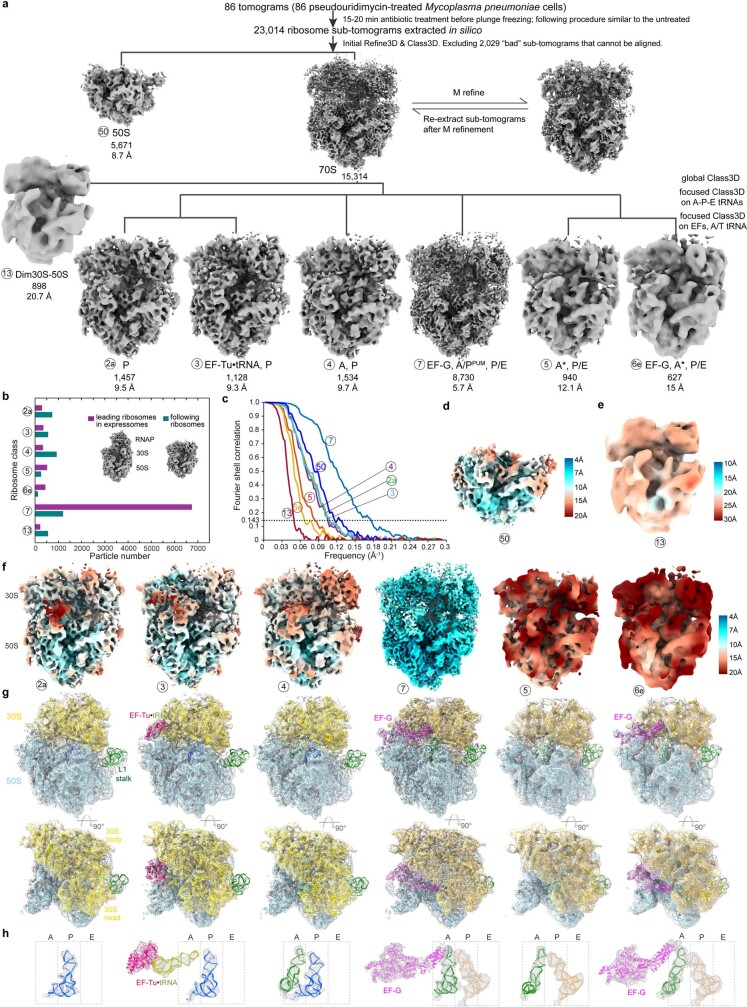
Classification, refinement and modelling of ribosomes in PUM-treated cells. **a**, Image-processing workflow for the PUM-treated dataset. The sub-tomogram classification and refinement are similar to those developed for the untreated dataset (Extended Data Fig. 4a). **b**, Distribution of translation states for ribosomes collided with a PUM-stalled RNA polymerase (purple; stalled expressome) and for the remaining ribosomes (green) in the PUM-treated cells. **c**, FSC curves for all classes calculated after refinements. **d**-**f**, Local-resolution maps. **g**, Models built for PUM-treated 70S classes. **h**, Elongation factors and tRNAs in the six 70S ribosome classes.

The ribosome is one of the most important targets for antibiotics, many of which are known to stall translation by stabilizing certain intermediates in the process^39^. To investigate the effects of antibiotics on the cellular translation machinery, we analysed ribosomes in cells that were treated for a short time (15–20 min; Methods) with two representative ribosome-targeting antibiotics: chloramphenicol (Cm), which binds to the peptidyl transfer centre in the 50S subunit and inhibits peptide bond formation^40,41^; and spectinomycin (Spc), which binds to the 30S neck and blocks translocation^14,28,42^. Overall, our sub-tomogram analysis of antibiotic-treated cells results in 17 ribosome maps, and shows that translation landscapes are markedly reshaped by different antibiotics (Fig. 3a, Extended Data Figs. 9–11, Supplementary Tables 4–6 and Supplementary Discussion).

In Cm-treated cells, 72% of 70S ribosomes are trapped in the 'A, P' state (class 4), owing to the inhibition of peptidyl transfer^39^ (Fig. 3a–d, Extended Data Fig. 9 and Supplementary Table 4). The Cm molecule was resolved in its canonical binding site^40,41^ (Extended Data Fig. 9d,g). Of note, 28% of ribosomes were found in several other elongation states, either before or following the major 'A, P' state (Fig. 3a and Extended Data Fig. 9). The existence of an EF-Tu•tRNA-bound state (class 3) is reminiscent of the effects of other antibiotics that inhibit peptidyl transfer in a similar manner^43–45^.

Spc is suggested to inhibit translocation by blocking head swivel of the 30S subunit^14,28,42^. We found that 67.5% of 70S ribosomes were stalled in the 'EF-G, A/P^Spc^, P/E' state in Spc-treated cells, a state similar to the pre-translocational 'EF-G, A/P, P/E' state (class 7) in untreated cells (Fig. 3a–d, Extended Data Fig. 10 and Supplementary Table 5). The Spc molecule density is clearly visible near helix 34 within the 30S neck region (Fig. 3b,c and Extended Data Fig. 10d,g), consistent with its reported binding site^14,42^. These results show that Spc inhibits translocation by impeding the dynamics of the 30S subunit. Similar to Cm, ribosomes in three additional elongation states could be detected with lower frequencies (Fig. 3a and Extended Data Fig. 10e,f).

Perturbation of other molecular pathways that are functionally coupled to translation in bacterial cells also affects the translation landscape; RNA polymerase stalled by pseudouridimycin (PUM) can physically block mRNA translocation in the ribosome that collides with it during transcription–translation coupling^2^. Consistently, 59.5% of 70S ribosomes in PUM-treated cells were found in the 'EF-G, A/P^PUM^, P/E' state (Fig. 3a, Extended Data Fig. 11 and Supplementary Table 6), which resembles the pre-translocational 'EF-G, A/P, P/E' state in untreated cells and the stalled 'EF-G, A/P^Spc^, P/E' state in Spc-treated cells (Fig. 3d). Our finding that physically obstructing mRNA translocation by a PUM-stalled RNA polymerase and chemically impeding 30S head dynamics by Spc lead to similar structures further confirms that mRNA translocation and 30S rotations are directly coupled.

The observation that treatment with antibiotics resulted in minor states that are not expected from their specific binding prompted us to investigate possible cell-to-cell variability in response to the antibiotics. To this end, we performed clustering analysis on the basis of translation elongation profiles in 577 tomograms of single cells under native and antibiotic treatment conditions. The analysis resulted in four major clusters in accordance with the four treatment groups, demonstrating small cell-to-cell variability within each cluster (Fig. 3e). Thus, our results show that the translation landscapes in cells are globally reshaped by small molecules specifically binding to ribosomes, as well as to other targets (Supplementary Discussion and Supplementary Table 8). The presence of minor elongation states under antibiotic treatment is reminiscent of previous studies that have shown ongoing slow translation in antibiotic-treated cells and context-dependent inhibition^46–48^. For example, Cm inhibition is affected by specific residues of the nascent peptide^46,47^. Most antibiotics, including Cm and Spc, are known to inhibit cell growth but do not immediately kill the cell^49^. It is possible that the reshaped translation landscapes by antibiotics lead to an imbalance in protein synthesis, which in the long run has detrimental consequences for the cell.

## Spatial and functional organization of translation

**Fig. 4:**
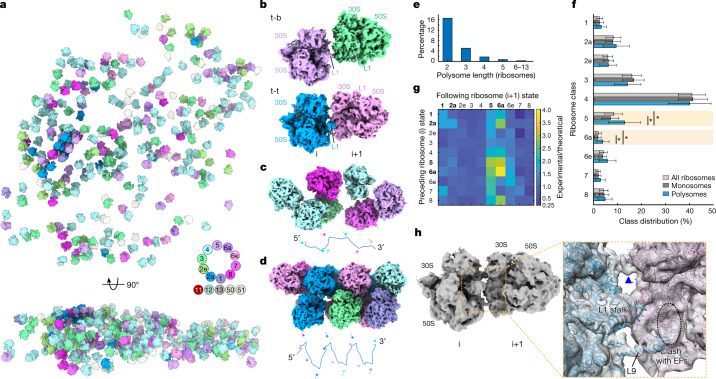
Spatial and functional organization of ribosomes in native cells. **a**, Three-dimensional map of ribosomes in a representative native untreated cell (selected from 356). Top, *x*–*y* view; bottom, orthogonal view. The 70S intermediates within the translation elongation cycle (classes 1–8, as in Fig. 2a), and additional classes (classes 11–13 and 50–51 as detailed in Extended Data Fig. 4) are coloured as indicated in the colour scheme (inset). 50S: light grey. **b**, 'top-back' (t-b) and 'top-top' (t-t) assembly patterns of adjacent ribosomes in polysomes. **c**,**d**, Representative long polysomes of loose (**c**) and tight (**d**) topologies, with the corresponding putative mRNA paths and nascent chain vectors shown underneath (not drawn to scale). **e**, Distribution of polysome lengths. **f**, Distribution of elongation states in polysomes compared to all ribosomes and mono-ribosomes. Bars and whiskers are mean and s.d. across 356 tomograms of untreated cells (*n* = 356 cells). Highlighted are states for which the fraction in polysomes differs by more than 50% compared to all ribosomes or mono-ribosomes. Asterisks indicate false discovery rate (FDR)-adjusted *P* value (*P*_FDR_) < 0.01 (two-sided Wilcoxon rank sum test). *P*_FDR_ values for class 5: 6.44 × 10^−23^ (polysome versus all) and 2.76 × 10^−39^ (polysome versus monosome); class 6a: 3.06 × 10^−28^ and 1.44 × 10^−34^, respectively. **g**, Occurrence frequencies of elongation state pairs of adjacent ribosomes in polysomes normalized to the theoretical probability of random pairs. States that require elongation factor binding to proceed are 1, 2a, 5 and 6a (in bold). **h**, Map of a di-ribosome within polysomes shows the intervening mRNA density (inset: blue arrowhead) and the extended L9 of the preceding ribosome (i). The C-terminal domain of extended L9 can interfere with elongation factor (EF) binding to the following ribosome (i+1).

**Extended Data Fig. 12:**
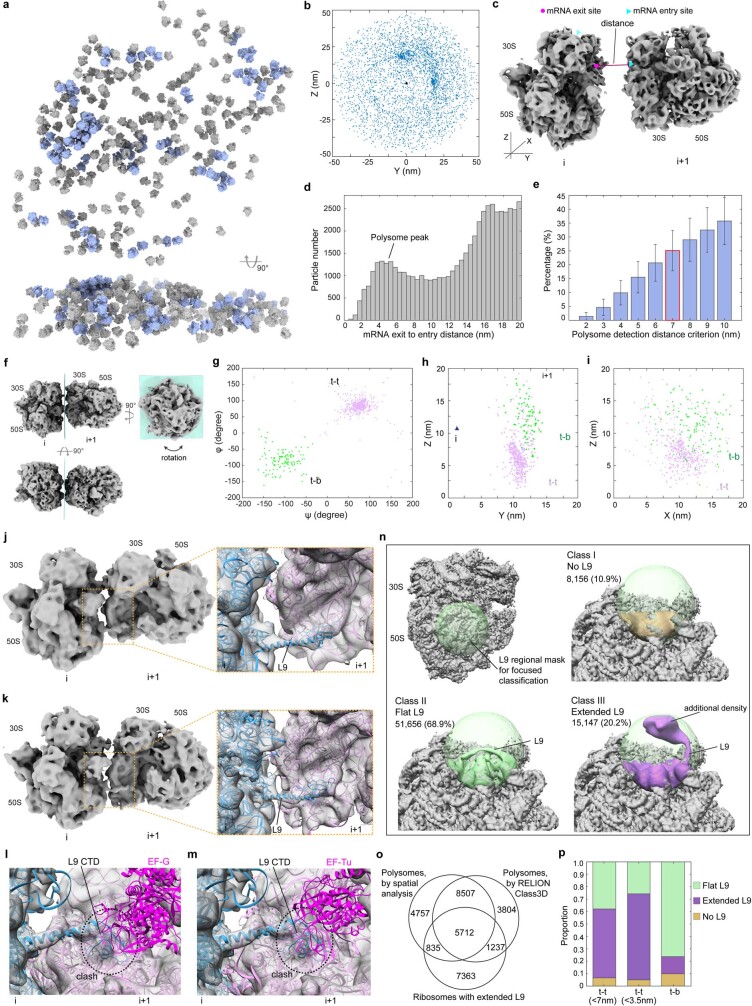
Spatial analysis of ribosomes and polysomes in native untreated cells. **a**, 70S ribosomes (grey) and detected polysomes (light blue) in a representative tomogram. **b**, Distribution of neighbouring ribosomes within a 50 nm centre-to-centre distance. Rotation normalized. **c**, Illustration of the polysome detection approach based on the distance from the mRNA exit site of one ribosome to the mRNA entry site of the next. **d**, Histogram of distances from the mRNA exit site of one ribosome to the mRNA entry sites of all neighbouring ribosomes. **e**, Percentages of ribosomes detected as polysomes using different distance thresholds (d). Mean and s.d. are across 356 tomograms (n = 356 cells). A 7 nm threshold was selected. **f**, In polysomes, the following ribosome (i) adopts various orientations relative to the preceding ribosome (i+1), mainly with regard to rotation around a plane perpendicular to the preceding ribosome’s mRNA exit site. **g**, Rotations of the following ribosome relative to the preceding ribosome in polysomes. The two clusters correspond to the previously defined 't-t' and 't-b' configurations. **h**-**i**, Positions of the following ribosomes (coloured according to different rotations) relative to the preceding ribosome (triangle indicates the mRNA exit site). **j**-**k**, Two representative di-ribosome pairs in polysomes with extended L9. **l**-**m**, Clash between the C-terminal domain of the extended L9 and the superposed EF-G and EF-Tu, respectively. **n**, *De novo* focused classification on the L9 region using a spherical mask (light green) of 70S ribosomes resulted in three classes: no L9 resolved (I), flat L9 (II) and extended L9 (III). The additional density connecting to the extended L9 originates from the neighbouring ribosome. **o**, Overlap between polysomes independently defined based on spatial analysis, RELION classification for polysomes and classified ribosomes with the extended L9. **p**, The extended L9 is more frequent in the 't-t' ribosome pairs, especially in compacted ones.

Finally, we investigated the spatial organization of active translation in native *M.* *pneumoniae* cells (Fig. 4a and Supplementary Discussion). It is known that ribosomes translating on the same mRNA can assemble closely in space to form polysomes^4,19^. We defined polysomes using a distance cut-off of 7 nm from the mRNA exit site to the mRNA entry site between neighbouring ribosomes (Extended Data Fig. 12a–e, Methods and Supplementary Discussion). The detected polysomes account for 26.2% of all 70S ribosomes, and two arrangement patterns between neighbouring ribosomes can be defined (Fig. 4b–e and Supplementary Discussion): the so-called 'top-top' (t-t; 78.5%, mRNA exit-to-entry distance 4.2 ± 1.4 nm) and 'top-back' (t-b; 21.5%, 5.4 ± 1.5 nm) configurations^4^. We also observed various topologies for long polysomes, ranging from loose assembly to tight packing with helix-like configurations^4,19,50^ (Fig. 4c,d).

**Extended Data Fig. 13:**
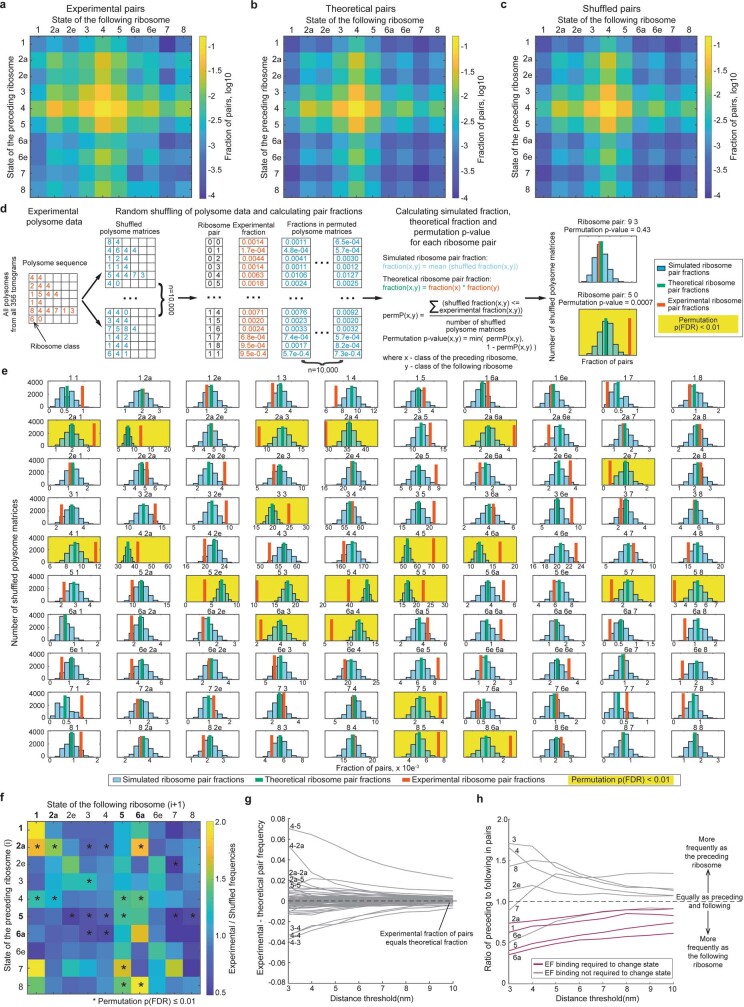
Correlation of translation elongation states within polysomes in native untreated cells. **a**, Occurrence frequencies of elongation state pairs of two adjacent ribosomes (preceding ribosome i versus following ribosome i+1) within polysomes calculated from the experimental data. **b**, Theoretical frequencies of elongation state pairs if there is no cross-influence within the polysome and ribosome state pairs form randomly. **c**, Frequencies of elongation state pairs calculated after random shuffling of the experimental data. **d**, Schematic representation of polysome shuffling analysis and permutation *p*-value calculation. Detailed procedure can be found in the Materials and Methods. **e**, Comparison of the experimental and shuffled pair fractions for all major pairs. **f**, Fold changes between the experimental and shuffled pair fractions from the shuffling experiments. Ribosomes of states that need elongation factor binding to proceed (states 1 and 2a for EF-Tu, and states 5 and 6a for EF-G) are more frequently engaged as the following ribosomes. Permutation *p*-values were adjusted for multiple hypotheses testing with the Benjamini–Hochberg procedure, and are provided in Supplementary Table 7. **g**, Difference between experimental and theoretical pair frequencies when using different distance thresholds for polysome definition. **h**, Ratios of the number of ribosomes in each state being the preceding one against the following one in polysome pairs (across all polysomes), calculated using different distance thresholds to define polysomes. Symmetric engagement as the preceding and following ribosome results in a ratio of 1.

Whether translation elongation is synchronized or coordinated within polysomes is a long-standing question^4,19,50^. To address this, we first examined whether the distribution of elongation states differs between the total ribosome population and polysomes. We found that although most states occurred equally frequently in both populations, the fractions of two states before EF-G binding (class 5, 'A*, P/E' and class 6a, 'A/P, P/E') are more frequent in polysomes (Fig. 4f). We next calculated the frequencies of state pairs between two adjacent ribosomes (preceding versus following) and compared them to theoretical pair frequencies calculated from the bulk distribution (Extended Data Fig. 13a–c). This comparison revealed that the occurrence of states among preceding and following ribosomes is not symmetric: following ribosomes more frequently populate states that require elongation factor binding to proceed to the next state (classes 1, 2a, 5 and 6a; Fig. 4g). We statistically validated such differences with a permutation test, and further show that the asymmetry between preceding and following ribosomes increases as the distance threshold used to define polysomes decreases (Extended Data Fig. 13d–h, Methods and Supplementary Discussion). This suggests that local coordination of translation elongation between adjacent ribosomes within the polysome is likely to be achieved by obstructing elongation factor binding that is required for the following ribosome to proceed to the next elongation state.

To investigate whether local coordination arises from structures specific to ribosomes engaged in polysomes, we performed structural classification of ribosome pairs to better resolve the ribosome–ribosome interface (Fig. 4h and Extended Data Fig. 12j,k). We found that within the interface of tightly packed polysomes, the ribosomal protein L9 of the preceding ribosome adopts an extended conformation and its C-terminal domain protrudes into the elongation-factor-binding site of the following ribosome (Fig. 4h and Extended Data Fig. 12j–m). Independent focused classification on L9 of all ribosomes showed that it mainly (68.9%) adopts a flat conformation on the ribosome surface, whereas it is extended in 20.2% of 70S ribosomes (Extended Data Fig. 12n). Ribosomes with the extended L9 largely overlap with the ribosomes detected as polysomes, especially those with a tighter 't-t' arrangement (Extended Data Fig. 12n–p). These results suggest that L9 tends to have a flat conformation in single ribosomes and to adopt the extended conformation within tightly assembled polysomes. This clarifies previous observations of L9 being extended in X-ray crystallography structures in which the crystal packing recapitulates configurations of compacted polysomes, but being found in the flat conformation in cryo-EM structures of isolated ribosomes^51^. Although L9 has been reported to be non-essential, its mutations can cause increased frameshifting and ribosome compaction by one codon^52,53^. We therefore propose that within tightly assembled polysomes, L9 of one ribosome can adopt an extended conformation that sterically interferes with elongation factor binding to the following ribosome. This local coordination mechanism can buffer adjacent ribosomes and help to maintain translation fidelity by avoiding direct collision within polysomes during active translation elongation.

## Conclusions

Our study demonstrates the potential of cryo-ET in shedding light on dynamic processes that are performed by macromolecular machines in living cells at a high level of detail. The analysis captures the structural and functional diversity of actively translating ribosomes inside a genome-reduced bacterium. The translation elongation cycle retrieved from the cellular cryo-ET data recapitulates and complements current mechanistic models of translation that are derived from controlled in vitro studies. The quantitative structural profiling of the cellular translation machinery presented here improves our understanding of protein biogenesis by providing distribution probabilities of translation intermediates. It also reveals how the translation machinery that functions as an interconnected system responds to different antibiotic perturbations on the single-cell level. Our investigation of polysomes further illustrates the advantage of in-cell structural biology, which can relate functional states of a molecular machine to its molecular sociology and cellular context, leading to the discovery of an elongation coordination mechanism mediated by L9. The approaches developed here establish a framework to analyse structural dynamics of cellular processes in the future and will contribute to the construction of functional cell models at atomic detail.

## Methods

### Cryo-ET sample preparation and data collection

The *M.* *pneumoniae* cultivation, sample preparation and data collection were described previously^2^. The three datasets of native untreated, Cm-treated and PUM-treated cells were re-processed in Warp and M 1.0.7 (the alpha versions that were officially released as v.1.0.9.)^3,54^. A small dataset of 15 tomograms acquired with Volta phase plate was processed with the denoising network in Warp 1.0.9 for visualization purposes only (as shown in Fig. 1a).

A dataset of spectinomycin-treated cells was collected following the same procedure as before^2^. In brief, spectinomycin (Sigma-Aldrich) at a final concentration of 0.4 mg ml^−1^ was added into the culture medium, 15–20 min before plunge-freezing. Tilt-series collection with the dose-symmetric scheme^55^ was performed on a Titan Krios transmission electron microscope equipped with a K3 camera (Gatan) using SerialEM 3.8 (ref. ^56^) with the following parameters: magnification 81,000×, pixel size 1.053 Å, tilt range −60° to 60° with 3° increment, total dose 120–140 e^−^ Å^−2^.

In total, 356 native untreated, 65 Cm-treated, 86 PUM-treated and 70 Spc-treated cellular tomograms were analysed in this work. Details of cryo-EM data collection, refinement and validation statistics are provided in Supplementary Tables 1 and 3–6.

### Image processing, ribosome template matching and map refinement

Pre-processing (motion correction, CTF estimation, dose filtering and tilt-series sorting) was performed in Warp 1.0.9 (ref.^3^). For the untreated, Cm-treated and PUM-treated datasets, the ribosome coordinates were adopted from previous particle picking^2^. For the Spc-treated dataset, template matching was performed in PyTom^57^, followed by computational classification in RELION 3.0 (refs.^58^,^59^ to exclude false positives, without manual cleaning. In total, 109,990 untreated, 21,299 Cm-treated, 23,014 PUM-treated and 13,418 Spc-treated ribosome sub-tomograms were reconstructed in Warp.

Three-dimensional (3D) refinement and classification were performed in RELION 3.0 (refs. ^58,59^). We then used the software M (v.1.0.9) to perform multi-particle refinement of the tilt-series and refine the average map^3^. Refinement of both geometric (image and volume deformation) and CTF parameters was done for five rounds in a sequential manner. After M refinement on the 70S, we performed focused refinement on the 30S and 50S subunits separately to improve the local map quality. Fourier shell correlation (FSC) calculation between independently refined random half subsets, local resolution estimation and post-processing were done in M and RELION.

### Atomic model building in high-resolution ribosome maps

Atomic models for the 30S and 50S subunits were built de novo using maps from focused refinement of Cm-treated ribosomes reported in our previous work^3^, which were deposited in the Electron Microscopy Data Bank (EMDB) under the accession codes EMD-11998 and EMD-11999. Homology models for *M.* *pneumoniae* ribosomal proteins were generated using the SWISS-MODEL^60^ online server (https://swissmodel.expasy.org/; accessed April 2020), with a *Bacillus subtilis* ribosome (Protein Data Bank (PDB) 3J9W) as the template, except for the ribosomal proteins L9 (PDB 1DIV and 4V63), L10 (PDB 1ZAV) and S21 (PDB 5MMJ). rRNA sequences (REFSEQ NC_000912) were aligned to the *E.* *coli* rRNA (PDB 4YBB) using the SINA online server^61^. ModeRNA^62^ was used to build homology models for rRNAs on the basis of the alignment, with PDB 4YBB as the template. Homology models were rigid-body-fitted into the cryo-ET densities using Chimera^63^, followed by iterative refinement using PHENIX real-space refinement^64^ and manual adjustment in Coot^65^. The ribosomal proteins L9, L10 and L11 were only fitted as rigid bodies into the map owing to the less-resolved local density. The flat conformation of L9 that resides on the ribosome surface was found to be predominant according to the focused classification (see Extended Data Fig. 12n) and was thus built in the model. Sequence extensions for ribosomal proteins S6, L22 and L29 were built de novo. Models were validated using MolProbity^66^. FSC curves between the model and the map were also calculated for validation^67^.

### Bioinformatic analysis of ribosomal proteins

Bioinformatic sequence analysis of ribosomal proteins in *M.* *pneumoniae* and the comparison with *E.* *coli* homologues were performed as follows, with the different steps described in detail below: (i) protein sequences and RefSeq genome annotation for the *M.* *pneumoniae* strain M129 (ATCC 29342) were downloaded from NCBI; (ii) protein sequences were also annotated with eggNOG-mapper to obtain COG (Clusters of Orthologous Groups) IDs^8,68^; (iii) for the annotated ribosomal proteins, the corresponding COG multiple sequence alignments from representative bacterial species were downloaded from the eggNOG database^69^; (iv) as *M.* *pneumoniae* M129 is not among the representative species, its protein sequences were added to the multiple sequence alignments with MAFFT software^70^; (v) for each COG multiple sequence alignment, the number of amino acids in every representative species (including *M.* *pneumoniae* M129) present at positions before the N terminus and after the C terminus positions of *E.* *coli* K-12 substr. MG1655 were calculated; (vi) the presence of N- or C-terminus extensions longer than 20 amino acids was illustrated in iTOL using NCBI taxonomy tree as the basis^71^; (vii) for all COGs corresponding to proteins with N- or C-terminus extensions in *M.* *pneumoniae* that are longer than 20 amino acids, protein disorder and secondary structure were analysed for all representative strains. The presence of cross-links or transposon insertions was also analysed.

RefSeq genome annotation and protein sequences for the *M.* *pneumoniae* strain M129 (ATCC 29342) were downloaded from NCBI for the *M.* *pneumoniae* M129 GCF_000027345.1 assembly from https://ftp.ncbi.nlm.nih.gov/genomes/all/GCF/000/027/345/GCF_000027345.1_ASM2734v1/ (accessed 21 April 2021). The file GCF_000027345.1_ASM2734v1_genomic.gff was used to identify ribosomal proteins annotated by RefSeq. The file GCF_000027345.1_ASM2734v1_protein.faa with protein sequences in FASTA format was used as input to the online tool eggNOG-mapper v2, which uses precomputed eggNOG v5.0 clusters and phylogenies for fast orthology assignment^69,72^. The annotation tables by RefSeq and eggNOG-mapper were merged by protein ID. In total, 52 ribosomal proteins were annotated and mapped to 51 unique COGs^68^. Two proteins, WP_010874426.1 and WP_010874827.1, were mapped to the same COG0267 corresponding to the ribosomal protein L33. Multiple sequence alignments for the annotated proteins from representative bacterial species were downloaded from the eggNOG v5.0 database^69^. For each of the 51 COG IDs mapping to ribosomal proteins in *M.* *pneumoniae*, the trimmed alignments were downloaded from http://eggnogapi5.embl.de/nog_data/text/trimmed_alg/COG_ID ('COG_ID' in the url should be changed to the corresponding COG ID). As *M.* *pneumoniae* M129 is not among the imported representative species, its sequences were added to the multiple sequence alignments. The sequence of each of the proteins was saved in a separate fasta file, and the MAFFT software v.7.475 was used in the add mode for each alignment as follows: mafft --reorder --add protein.fasta --auto trimmed_alg_COG.fa > output_file (ref. ^70^). For COG0627, the protein WP_010874827.1 from *M.* *pneumoniae* was used.

For each of the ribosomal COGs, the amino acid positions of the protein from the *E.* *coli* strain K-12 substr. MG1655 was taken as a reference, and for each protein from representative species (including *M.* *pneumoniae* M129), the number of amino acids before the N-terminus and after the C-terminus locations in *E.* *coli* was calculated. In total, 11 ribosomal proteins that had N- or C-terminus extensions of more than 20 amino acids in *M. pneumoniae* M129 were considered to have extensions (2 proteins with N-terminus extensions and 9 proteins with C-terminus extensions). Ribosomal protein S3 (COG0092), which has a 17-amino-acid extension at the C terminus, was also retained for further analysis. NCBI bacterial taxonomy file new_taxdump.zip was downloaded from https://ftp.ncbi.nlm.nih.gov/pub/taxonomy/new_taxdump/ (version on 24 April 2021). The file names.dmp was used to map NCBI IDs to taxonomy names. Tree was reconstructed from the file taxidlineage.dmp in Python v.3.7.7 with ETE3 Toolkit v.3.1.2 (ref. ^73^). EggNOG protein IDs containing NCBI species IDs were directly mapped to the NCBI tree nodes. The presence of ribosomal protein extensions was saved as tables and converted to iTOL format with table2itol utility (https://github.com/mgoeker/table2itol) in R v.3.6.1. The tree was visualized with iTOL v.6 (ref. ^71^).

Sequences for the 11 ribosomal proteins annotated with extensions in *M.* *pneumoniae*, and their orthologues in other species, were further analysed in terms of protein disorder and secondary structures. For disorder prediction, all protein sequences from the multiple sequence alignment files were saved in FASTA format without gaps. The IUPred2A tool in Python v.3.7.7 was run with the input parameter 'long' for each protein sequence^74^. The number of disordered amino acids and disorder length in extended regions were calculated on the basis of the position of the extended region relative to *E.* *coli*, and an IUPred2A score of 0.5 was used as the disorder threshold. The JPred prediction tool^75^ was used to predict the secondary structure of each protein using the jpredapi utility and JPred-big-batch-submission utility for a large number of submissions (https://github.com/fabianegli/JPred-big-batch-submission). The number of helices in extended regions was calculated on the basis of the position of the extended region relative to *E.* *coli* protein.

For the 11 ribosomal proteins with extensions in *M.* *pneumoniae*, the positions of cross-links linking to the same protein or a different protein were mapped according to the amino acid cross-link positions reported previously^2^. To analyse transposon insertions in *M.* *pneumoniae* ribosomal proteins with extensions, the supplementary materials from a previous publication^24^ were used. All the files in the 'SupplementaryData1_fastqins_processed.zip folder' (files with extension '.qins' but not '_fw.qins' or '_rv.qins') were concatenated to obtain a list of nucleotide coordinates of transposon insertions in the sequence of *M.* *pneumoniae* M129 NC_000912.1. The transposon insertion locations in the 11 ribosomal proteins were selected on the basis of their genomic locations as per RefSeq annotations (GCF_000027345.1_ASM2734v1_genomic.gff). Nucleotide locations were converted to amino acid locations by calculating the difference between each transposon location and the start coordinate of the corresponding gene and dividing the number of nucleotides by three. All data were mapped to the protein sequences to derive Extended Data Fig. 3.

### Sub-tomogram classification of the translation states of ribosomes

Maximum-likelihood 3D classification^76^ was performed in RELION 3.0 (refs. ^58,59^) with the re-extracted ribosome sub-tomograms after M refinement. A hierarchical and exhaustive classification strategy (Supplementary Discussion) with at least three tiers was used to handle the heterogeneity in the native untreated dataset, which is described here in detail and illustrated in Extended Data Figs. 4 and 5. A similar procedure was applied for all antibiotics-treated datasets, and is detailed in Extended Data Figs. 9–11.

In the first tier, 109,990 sub-tomograms were classified into 70S and free 50S with a global spherical mask (320-Å diameter). The 24,157 free 50S were further classified into two classes, with a local spherical mask focusing on the ribosome recycling factor binding site. Before extensive 70S classification, structural heterogeneity was evaluated by visual inspection, multibody refinement and test classification runs in RELION. Classification set-ups were extensively tested, including different masks (global 70S mask, local 30S mask, spherical tRNA path mask, solvent tRNA path mask, spherical EF mask, solvent EF mask), initial references (ribosome average, features-less sphere or none), angular search options (global, local or without alignment) and RELION optimization parameters (class numbers 2–16, T values 2–10, iterations 25–40, limit resolution E-step 5–10 Å). To avoid bias, we mostly used a sphere or other featureless shapes with soft edges as the mask. Local spherical masks covering factors (elongation factors and/or tRNAs) generally provided more consistent and stable classification results than those obtained when using larger masks for the untreated data. The class number was made higher than the number of distinct structures that could be retained in one classification job and similar resulting classes were grouped.

In the second tier, the 77,539 identified 70S ribosomes were further classified with a local mask (Extended Data Fig. 4a, mask I) focusing on the tRNA path region, roughly covering the A, P and E sites. This resulted in four major classes with different tRNA occupancies: 'P, E', 'a, P/E', 'P' and 'A, P'. In the following rounds, we could further classify the 'a, P/E' class into 'A*, P/E' and 'A/P, P/E'. In addition, a 70S class with only one hybrid 'P/E' tRNA was classified. Two of the resulting classes were not interpretable: 2,150 particles with dim 30S density that were poorly resolved and 1,484 particles with density near the P site that does not resemble a tRNA.

In the third tier, focused classification with a local mask (Extended Data Fig. 4a, mask II) around the elongation factor and A/T tRNA binding sites was carried out. For the previous class with 'P, E' tRNAs, further classification resulted in 1,803 particles without additional density, 3,324 particles with EF-G (updated as 'EF-G, ap/P, pe/E') and 4,634 particles with EF-Tu•tRNA. For the classes with partial and full hybrid tRNAs, sub-classes with EF-G were obtained. For the class with only 'P' tRNA, 12,464 particles with additional EF-Tu•tRNA were classified.

For each classification step, at least three parallel RELION classification jobs with the same or slightly different parameters (either 30S mask or spherical EF/tRNA mask, local angular search range, class number, T value) were carried out for comparison. The classification job with the most stable result was selected and used for sorting sub-tomograms (Extended Data Fig. 5a–g). After sorting, subsequent validation classification runs were performed for each sorted class to test whether new structures emerge or whether particles were 'wrongly' classified. Misclassified particles were relocated to the corresponding class and the validation runs were repeated until convergence. This approach was performed for all classification steps. For each of the final classes, refinement and post-processing were done in RELION. Further classification performed with the new refinement results as inputs did not generate any new classes.

### Model building and comparison of the ribosome translation states

To build models for the ribosome classes, the 30S and 50S models built as described above were used as starting models for flexible fitting. Homology models of EF-G and EF-Tu were generated by SWISS-MODEL (https://swissmodel.expasy.org/; accessed April 2020) with PDB structures 4V7D and 4V5L as the template, respectively. For tRNA homology models, the tRNA in an *E.* *coli* ribosome structure (PDB 4V7C) was used as the template and mutated to the sequence of *M.* *pneumoniae* Phe-tRNA (REFSEQ NC_000912). The mRNA and nascent peptide were built using PDB 3J9W as the template. The homology model of the ribosome recycling factor was built using PDB 1EH1 as the template. For each class, the starting models were first rigid-body-fitted into the density using Chimera and then flexible fitting was done using Namdinator^77–79^. Validation was performed as described above.

For measuring 30S body rotations, structures of all classes were first aligned to the 50S subunit and then the rotation angles were estimated with a pivot point near nucleotide 11 of the 16S rRNA. With the perspective from the solvent side of the 30S subunit, positive numbers equal counter-clockwise rotations and negative numbers equal clockwise rotations. For measuring 30S head rotations, all class structures were first aligned to the 30S body and then the rotation angles were determined with the axis near the 30S neck. For both rotations, the angles in the 'A, P' class were defined as 0°. To describe the L1 dynamics, the distance between the mass centre of the L1 stalk (near nucleotide 2,181 of the 23S rRNA) and a fixed point near the centre of the classical E site (determined on the basis of the 'P, E' class) was measured after aligning all classes on the 50S subunit (excluding the L1 stalk).

### Spatial analysis of ribosomes and polysomes

Spatial mapping of ribosomes within cellular tomograms was achieved by projecting back the ribosome structures into the tomograms, with coordinates determined by template matching and shifts and rotations determined by RELION refinement. The projection was performed using the TOM toolbox^80^ after Euler angle format conversion, at four times binning (voxel size 6.8 Å). To calculate the ribosome concentration, we first estimated the cellular volume covered in the tomogram and then divided the total number of detected ribosomes by the volume.

Detection of the polysomes was based on both position and orientation information (Extended Data Fig. 12c), using a custom script in MATLAB 2016b. It is noted that the annotated polysomes only refer to those assembled closely in space and thus can be detected on the basis of their spatial proximity. The positions of mRNA entry and exit sites for all 70S ribosomes were calculated on the basis of the rotations and shifts determined during RELION refinement. The distances from the mRNA exit site of one ribosome (defined as the preceding ribosome i) to the mRNA entry sites of all neighbouring ribosomes (as potential following ribosomes i+1) were calculated. A distance threshold of 7 nm was used to define whether two ribosomes belong to the same polysome. The calculation and polysome definition were done for all 70S ribosomes. A unique identifier was assigned to each polysome, as well as the sequential number for all ribosomes within the polysome.

The distribution of relative positions of adjacent ribosomes within the polysome—that is, the position of the following ribosome (i+1) relative to the preceding ribosome (i)—was analysed after normalizing the relative position vectors with rotations of the preceding ribosome determined from RELION refinement. The relative rotation of the following ribosome to the preceding ribosome was represented as three Euler angles (*ψ*, *θ*, *φ* in XYZ system). Using the *k*-means clustering function in MATLAB 2016b, we determined two major arrangement configurations for adjacent ribosomes within the polysome; that is, how the following ribosome rotates relative to the preceding ribosome (as shown in Extended Data Fig. 12f,g). These two configurations are identical to the previously reported 'top-top' (t-t) and 'top-back' (t-b) configurations^4^, and the naming was adopted.

To further refine the ribosome–ribosome interface in polysomes, RELION classification was performed with sub-tomograms extracted with a large box size that can accommodate two ribosomes. After refinement on the preceding ribosome (i), classification without refinement was performed with a local mask focusing on the following ribosome (i+1). Only ribosome pairs within tightly packed polysomes (5,083 pairs from 5 di-ribosome classes; all with a 't-t' arrangement and extended L9 in between) resulted in average densities with both ribosomes well resolved. Models for the resulting classes were generated with rigid-body-fitting of the above described  ribosome models and the L9 homology model with the extended conformation (PDB 4V63).

### Statistical analysis of translation elongation states in polysomes

To compare the experimental elongation state frequencies in polysomes with theoretical frequencies, the distributions of frequencies of each elongation state calculated across 356 tomograms of the untreated cells were compared between polysomes and all ribosomes, and between polysomes and mono-ribosomes by calculating the fold change between distribution medians. Statistical significance was assessed with a two-sided Wilcoxon–Mann–Whitney test using the ranksum function in MATLAB 2019b. The theoretical frequency of each ribosome pair was calculated as the product of the overall frequencies of the ribosome classes for the preceding ribosome (i) and the following ribosome (i+1). Experimental polysome pair frequencies were calculated by summarizing the numbers of all ribosome pairs engaged in polysomes, and dividing these numbers by the total number of pairs. Experimental and theoretical pair frequencies were compared by calculating the fold change per pair.

Permutation analysis was performed to test the significance of differences between occurrence frequencies of the experimental pairs compared to the theoretical pairs. The ribosome pair sequences in polysomes were represented as a matrix with single polysomes as rows and ribosome positions as columns, in which each matrix cell contains the ribosome class representing its elongation state. Columns with positions beyond each ribosome’s length were assigned to NaN (not-a-number). All polysomes across all tomograms were combined in one matrix (8,641 polysomes in total). For permutation analysis, all elements of the polysome matrix were randomly shuffled for 10,000 times with the randperm function in MATLAB 2019b. For each row, the elements were sorted so that the ribosome classes are in the front columns followed by NaNs occurring in the same row after shuffling. Rows with fewer than two ribosomes in the sequence were deleted. Shuffled ribosome pair frequencies were calculated in the same way as experimental pair frequencies. The permutation *P* value for each ribosome pair was calculated as the minimum between the number of permutations in which the pair frequency was less or equal to the experimentally observed frequency divided by the total number of permutations, and the one minus this value. Permutation *P* values were adjusted for multiple hypotheses testing with the Benjamini–Hochberg procedure using the mafdr function in MATLAB 2019b with parameters ('bhfdr', 'true').

For polysome distance threshold analysis, matrices were created from the full ribosome dataset by varying the distance threshold to the nearest neighbour in the range from 3 to 10 nm. The distances for polysome definition were calculated between the mRNA exit site of one ribosome and the mRNA entry site of another ribosome as described above. The fractions of elongation states in ribosome pairs were calculated as for the original ribosome set (with the threshold of 7 nm). For each distance threshold and each ribosome class, the ratio between the number of pairs in which the preceding ribosome has this class and the number of pairs in which the following ribosome has this class was calculated.

### Single-cell clustering analysis

The distributions of 70S ribosome classes identified in the translation elongation phase for all four datasets (356 untreated cells, 65 Cm-treated cells, 70 Spc-treated cells and 86 PUM-treated cells) were used for clustering analysis. As each tomogram covers most of one cell, the sub-tomograms in each class could be further separated by which cell they belong to. Classes in the antibiotic-treated cells were assigned with the class identifiers according to the closest classes in the untreated dataset. The percentages of different classes within each cell were calculated and these numbers were used as inputs for clustering analysis. Hierarchical clustering analysis was done using the clustergram function in MATLAB 2016b.

Structure visualization, preparation for figures and videos were done in Chimera^63^ and ChimeraX^81^.

### Reporting summary

Further information on research design is available in the Nature Research Reporting Summary linked to this article.

## Online content

Any methods, additional references, Nature Research reporting summaries, source data, extended data, supplementary information, acknowledgements, peer review information; details of author contributions and competing interests; and statements of data and code availability are available at 10.1038/s41586-022-05255-2.

## Supplementary information

Supplementary Information This file contains Supplementary Discussion; Supplementary Tables 1–8; legends for Supplementary Videos 1-2 and Supplementary References.

Reporting Summary

Supplementary Video 1

Supplementary Video 2

## Data Availability

Maps have been deposited in the EMDB under accession codes 13234, 13272, 13273, 13274, 13275, 13276, 13277, 13278, 13279, 13280, 13281, 13282, 13283, 13284, 13285, 13286, 13410, 13411, 13412, 13413, 13414, 13431, 13432, 13433, 13434, 13435, 13436, 13445, 13446, 13447, 13448, 13449, 13450, 13451, 13452, 13287, 13288 and 13289. Models have been deposited in the PDB under accession codes: 7OOC, 7OOD, 7P6Z, 7PAH, 7PAI, 7PAJ, 7PAK, 7PAL, 7PAM, 7PAN, 7PAO, 7PAQ, 7PAR, 7PAS, 7PAT, 7PAU, 7PH9, 7PHA, 7PHB, 7PHC, 7PI8, 7PI9, 7PIA, 7PIB, 7PIC, 7PIO, 7PIP, 7PIQ, 7PIR, 7PIS and 7PIT. Detailed information for all maps and models generated in this work is provided in Supplementary Tables 1 and 3–6. Maps and atomic models used from previous studies were obtained from the EMDB (11998 and 11999) and the PDB (3J9W, 1DIV, 4V63, 1ZAV, 5MMJ, 4YBB, 4V7C, 4V7D, 4V5L and 1EH1).

## Code availability

The code and associated data for bioinformatics analysis of ribosomal protein extensions (Extended Data Fig. 3) and statistical analysis of polysome sequences (Fig. 4 and Extended Data Fig. 13) are deposited at GitHub (https://github.com/mszimmermann/mycoplasma_ribosome). The MATLAB script for polysome annotation (Fig. 4 and Extended Data Fig. 12) can be found at https://github.com/xueliang4906/polysome_detect.
