# Publisher Correction: Visualizing translation dynamics at atomic detail inside a bacterial cell

**DOI:** 10.1038/s41586-022-05455-w

**Published:** 2022-11-10

**Authors:** Liang Xue, Swantje Lenz, Maria Zimmermann-Kogadeeva, Dimitry Tegunov, Patrick Cramer, Peer Bork, Juri Rappsilber, Julia Mahamid

**Affiliations:** 1 Structural and Computational Biology Unit, European Molecular Biology Laboratory (EMBL), Heidelberg, Germany; 2 Collaboration for joint PhD degree between EMBL and Heidelberg University, Faculty of Biosciences, Heidelberg, Germany; 3 Chair of Bioanalytics, Technische Universität Berlin, Berlin, Germany; 4 Department of Molecular Biology, Max-Planck-Institute for Biophysical Chemistry, Göttingen, Germany; 5 Yonsei Frontier Lab, Yonsei University, Seoul, South Korea; 6 Department of Bioinformatics, Biocenter, University of Würzburg, Würzburg, Germany; 7 Wellcome Centre for Cell Biology, University of Edinburgh, Edinburgh, UK

**Keywords:** Cryoelectron tomography, Bacterial structural biology, Cryoelectron tomography, Ribosome

Correction to: *Nature* 10.1038/s41586-022-05255-2 Published online 28 September 2022

In the version of this article initially published, the orientation of Figure 3a (“+PUM” at top, “Untreated” at bottom) was incorrect and has been amended to read left-to-right, in alignment with Figure 3e, in the HTML and PDF versions of the article

