# Supplementary material for: Visualizing translation dynamics at atomic detail inside a bacterial cell
Source: Nature. 2022 Sep 28;610(7930):205–11. doi: 10.1038/s41586-022-05255-2 (PMC9534751; doi:10.1038/s41586-022-05255-2)
Supplement: Supplementary file 1 — This file contains Supplementary Discussion; Supplementary Tables 1–8; legends for Supplementary Videos 1-2 and Supplementary References. [file 41586_2022_5255_MOESM1_ESM.pdf]

nature portfolio

https://doi.org/10.1038/s41586-022-05255-2

Supplementary information

Visualizing translation dynamics at atomic detail inside a bacterial cell

In the format provided by the
authors and unedited

Supplementary Information for

Visualizing translation dynamics at atomic detail inside a bacterial cell

Liang Xue[1,2], Swantje Lenz[3], Maria Zimmermann-Kogadeeva[1], Dimitry Tegunov[4], Patrick Cramer[4], Peer Bork[1,5,6], Juri Rappsilber[3,7], Julia Mahamid[1*]

[1] Structural and Computational Biology Unit, European Molecular Biology Laboratory (EMBL), Meyerhofstraße 1, 69117 Heidelberg, Germany.

[2] Collaboration for joint PhD degree between EMBL and Heidelberg University, Faculty of Biosciences, Heidelberg, Germany.

[3] Technische Universität Berlin, Chair of Bioanalytics, 10623 Berlin, Germany.

[4] Department of Molecular Biology, Max-Planck-Institute for Biophysical Chemistry, Am Faßberg 11, 37077 Göttingen, Germany.

[5] Yonsei Frontier Lab, Yonsei University, Seoul 03722, South Korea.

[6] Department of Bioinformatics, Biocenter, University of Würzburg, Würzburg, Germany.

[7] Wellcome Centre for Cell Biology, University of Edinburgh, Max Born Crescent, Edinburgh, EH9 3BF, UK.

*Corresponding author: julia.mahamid@embl.de

Table of Contents

Supplementary Discussion **3**

Atomic ribosome model reveals divergent protein extensions 3

Ribosome classification for probing the translation process in cells 6

Translation landscapes in *M. pneumoniae* 13

Spatial analysis of ribosomes and polysomes 13

Coordination of translation elongation within the polysomes 14

Effects of antibiotics on the translation machinery 16

Supplementary Table 1 ..... **3**

Supplementary Table 2 ..... **5**

Supplementary Tables 3-6 ..... **8**

Supplementary Table 7 ..... **15**

Supplementary Table 8 ..... **17**

Supplementary Videos 1 & 2 ..... **18**

Supplementary References **19**

Supplementary Discussion

Atomic model of the *M. pneumoniae* ribosome reveals divergent protein extensions

Refinement of the 18,987 sub-tomograms containing 70S ribosomes from 65 Cm-treated cells, and refinement of the 77,539 sub-tomograms from 356 untreated cells both resulted in 70S ribosome maps with a nominal resolution of 3.5 Å (Extended Data Fig. 1a-f). Local resolutions of the 50S subunit in both datasets are at the Nyquist limit of 3.4 Å (Extended Data Fig. 1b-j). For the 30S subunit, focused refinement of the Cm-treated ribosomes generated higher resolution (3.7 Å) compared to the untreated dataset (Extended Data Fig. 1b-j), because of the reduced structural dynamics upon Cm binding. The better resolved densities from the Cm-treated dataset were used to build atomic models for the 30S and 50S subunits separately (Extended Data Fig. 1g-k). These were used as initial models to build models into the 70S ribosome average map from untreated cells (Methods, Supplementary Table 1).

Extensions of the eleven ribosomal proteins are all solvent-exposed on the ribosome surface and positioned away from the active sites (Fig. 1c, Extended Data Fig. 2e). They are therefore not expected to be directly involved in the translation process. However, the ribosomal proteins bearing these extensions are located near important interaction interfaces of the ribosome; our previous in-cell crosslinking mass spectrometry results[2] indicate that the extensions are crosslinked to different proteins (Extended Data Fig. 3a). Ribosomal proteins S2, S3 and S5 are located near the mRNA entry site on the ribosome, and have been suggested to form the main contact interface with the interacting RNA polymerase (RNAP) during transcription-translation coupling in *M. pneumoniae*[2]. S6 resides near the interface of the two ribosomal subunits, but its long C-terminal extension can extend towards the mRNA entry site. S6 is crosslinked to the delta subunit of the RNAP in the in-cell crosslinking mass spectrometry data (Extended Data Fig. 2f-g). These results suggest that extensions of 30S proteins may have functional roles near the mRNA entry site of the ribosome. On the 50S subunit, three (L22, L23 and L29) of the four ribosomal proteins surrounding the nascent peptide tunnel have extended sequences (Fig. 1c, Extended Data Fig. 2e, 3a). The long helices formed by the extensions of L22 and L29 mainly interact with peripheral rRNA. All three extensions are over 30 Å away from the nascent peptide exit site and do not appear to be able to interact with the nascent peptide during translation (Fig. 1c, Extended Data Fig. 2e). Although functions of the ribosomal protein extensions remain unknown, a transposon mutation screen in *M. pneumoniae* shows that their disruption affects cellular fitness or survival[24] (Extended Data Fig. 3a). Some disordered extensions of ribosomal proteins in other bacterial species have been suggested to play roles in ribosome biogenesis or assembly[82-85]. Ribosomal protein extensions were also recently suggested to be the main driving force for the formation and coevolution of protein interaction networks in ribosomes[83]. It is thus possible that rather than direct involvement in the translation process, ribosomal protein extensions play important roles in ribosome biogenesis and/or assembly in *M. pneumoniae*.

Supplementary Table 1 | Cryo-EM data collection, refinement and validation statistics of high-resolution ribosome averages.

	#1 50S subunit<br>in Cm-treated cells<br>EMD-11999<br>PDB 7OOD	#2 30S subunit<br>in Cm-treated cells<br>EMD-11998<br>PDB 7OOC	#3 70S ribosome average<br>in untreated cells<br>EMD-13234<br>PDB 7P6Z
**Data collection and processing**			
Magnification	81,000		
Voltage (kV)	300		
Electron exposure (e⁻/Å^2)	115 to 135		
Defocus range (μm)	-1.5 to -3.5		
Pixel size (Å)	1.7005		
Symmetry imposed	C1		
Initial particle images (no.)	21,299	21,299	109,990
Final particle images (no.)	18,987	18,987	77,539
Map resolution (Å)<br>FSC threshold 0.143	3.4	3.7	3.5
Map resolution range (Å)	3.4 to 6	3.4 to 8	3.5 to 8
Map sharpening B factor (Å^2)	-30 to -10	-30 to -10	-30 to -10
**Model refinement**			
Initial model used (PDB code)	3J9W, 1DIV, 4V63, 1ZAV, 4YBB	3J9W, 5MMJ, 4YBB	7OOC, 7OOD, 4V7C, 3J9W
Model resolution (Å)<br>FSC threshold = 0.143	3.4	3.7	3.6
Model vs. map correlation coefficient (cc_mask)	0.84	0.83	0.85
Model composition			
Non-hydrogen atoms	89,516	51,227	142,534
Protein residues	3,449	2,471	5,925
RNA residues	2,984	1,493	4,562
Ligands	30	2	30
B factors (Å^2) (mean)			
Protein	258.13	133.51	124.58
RNA	87.37	91.88	133.01
Ligand	97.18	112.70	77.30
R.m.s. deviations			
Bond lengths (Å)	0.010	0.003	0.003
Bond angles (°)	1.351	0.612	0.748
Validation			
MolProbity score	2.37	2.24	2.12
Clashscore	7.92	12.30	13.63
Poor rotamers (%)	3.23	1.45	0.0
Ramachandran plot			
Favored (%)	89.83	91.24	92.25
Allowed (%)	10.00	8.68	7.63
Disallowed (%)	0.18	0.08	0.12

Our bioinformatic analysis revealed that 1% to 78% out of the 4,396 tested representative bacteria strains have ribosomal protein extensions in comparison to *E. coli* K-12 (Extended Data Fig. 3, Supplementary Table 2). The largest frequency of extensions (78%) was detected in L4, including the extension in *M. pneumoniae* M129, although the sequence lengths are comparable between species. This possibly reflects the fact that the sequence at the C-terminus of L4 is different in Gammaproteobacteria compared to other bacteria, which results in multiple sequence alignment where many bacteria have a C-terminus extension compared to *E. coli*. For the other ten ribosomal proteins with extensions, the extensions are at least 20 amino acids longer in *M. pneumoniae*, compared to both *E. coli* and *B. subtilis*. These protein extensions, however, are not specific to any phylum or sub-groups of bacteria (Extended Data Fig. 3b). Within the Tenericutes phylum or the Mollicutes class to which *M. pneumoniae* belongs, the extensions appear to be more frequent than other bacteria. Moreover, extensions in some bacteria, such as *Mycobacterium smegmatis*[86-88], are not limited to the eleven ribosomal proteins found in *M. pneumoniae*. The observation that *M. pneumoniae* as genome-reduced bacterium that lives a strict parasitic lifestyle as a human pathogen[89] has many ribosomal proteins with extensions is reminiscent of reports that parasitic protozoans also exhibit extensive ribosomal protein extensions compared to other eukaryotes[90]. Structure determination of ribosomes from non-model species will be necessary to understand evolution and diversity of ribosomes in adaptation to different environments.

Supplementary Table 2 | Statistics of the eleven ribosomal proteins with extensions across bacteria.

Protein	S2	S3	S5	S6	S18	L3	L4	L22	L23	L29	L31
Length of extension in *M. p.* (amino acids)	46	17	57	74	30	74	50	49	139	47	25
Number of disordered residues (score>0.5) in *M. p.*	29 (63%)	17 100%	57 100%	67 (90%)	23 (76%)	68 (91%)	2 (4%)	46 (93%)	110 (79%)	11 (23%)	8 (32%)
Number of predicted helices in *M. p.*	19 (41%)	0 (0%)	1 (1%)	23 (31%)	0 (0%)	5 (6%)	15 (30%)	23 (46%)	0 (0%)	28 (59%)	0 (0%)
Number of predicted beta sheets in *M. p.*	0 (0%)	0 (0%)	0 (0%)	0 (0%)	2 (6%)	2 (2%)	10 (20%)	0 (0%)	0 (0%)	0 (0%)	0 (0%)
Number of species with extensions (out of 4,396)	1,956 (44%)	851 (19%)	895 (20%)	50 (1%)	396 (9%)	579 (13%)	3,438 (78%)	212 (4%)	44 (1%)	159 (3%)	138 (3%)
Number of species with >50% disordered residues in extensions	1,618 (82%)	840 (98%)	890 (99%)	16 (32%)	200 (50%)	501 (86%)	55 (1%)	193 (91%)	20 (45%)	150 (94%)	113 (81%)
Number of species with >50% helices in extensions	50 (2%)	0 (0%)	0 (0%)	2 (4%)	0 (0%)	0 (0%)	0 (0%)	84 (39%)	26 (59%)	42 (26%)	17 (12%)
Number of species with >50% beta sheets in extensions	0 (0%)	0 (0%)	0 (0%)	0 (0%)	0 (0%)	0 (0%)	0 (0%)	0 (0%)	0 (0%)	0 (0%)	0 (0%)

M. p.: *Mycoplasma pneumoniae*

Ribosome classification for probing the translation process in cells

The structural dynamics of ribosomes along their functional trajectories, as well as the distribution of these states, are expected to be preserved in a close-to-native condition in frozen-hydrated cells. The vitrification time[91] (0.1- 0.2 millisecond) is much faster than any known major translation steps[5,34,92] (at least at millisecond scale). *M. pneumoniae* cells in the fast-growing phase were directly grown on carbon-coated gold grids at 37 °C in rich medium, minimising potential perturbation prior to vitrification. Active translation is expected to be ongoing in *M. pneumoniae* cells right before plunge freezing. Vitrification was done with a manual plunger without a temperature controller. The cells on the grid were exposed to room temperature (20-25 °C) during the blotting step (~3 seconds). We cannot exclude the possibility that this short exposure led to a slight temperature change to cells on the grid.

A hierarchical and exhaustive classification procedure was designed to mitigate potential variations associated with individual RELION classification jobs (Methods, Extended Data Fig. 4, 5), and to ensure the proportions of classified translation states are representative of their distribution inside cells. We further validated unbiased representation of 70S ribosome localization and the robustness of the classification, by plotting the class distribution against template matching cross-correlation coefficients (Extended Data Fig. 5h, i). The extracted sub-tomograms retrieved the vast majority (>90%) of 70S ribosomes and a subpopulation (~50%) of free 50S in the cellular tomograms (Extended Data Fig. 5h). The analysis shows that there is no bias for different 70S classes due to template matching (Extended Data Fig. 5i). Thus, the obtained structures and their distribution represent a good approximation of the 70S ribosome population in native *M. pneumoniae* cells. We recognize that rare states, such as those in the initiation and termination phases, may not be identified as distinct classes[93]. The classes that were determined represent states that are more populated and structurally distinct. It is also possible they represent averages of ensembles of very close intermediate states[34].

The sub-tomograms in the most populated "A, P" class could not be further classified to distinguish states before and after peptidyl transfer. Although the nascent peptide density around the A site appears to be stronger compared to the P site, it is impossible to determine the exact state from the density alone (Fig. 2b). No class with all three A-, P- and E-site tRNAs was resolved, different from a number of *in vitro* structures[13,94,95]. Although our result does not rule out the possibility that ribosomes with all three A, P, and E site tRNAs exist in cells, they may represent transient intermediates with low occurrence frequencies. This agrees with a previous single-molecule study showing that ribosomes are rarely occupied (1.7%) by three tRNAs during active translation[31]. We could not identify any class with EF-G binding to non-rotated ribosomes, suggesting that they represent less energetically favourable states[38]. For the two classes with EF-Tu, the blurred local density indicates

the motion of EF-Tu during decoding[13,96]. Further classification with different focused masks and setups, however, did not result in any distinct sub-classes.

In addition to the ten elongation classes, we captured five classes that may represent states in other translation phases (Extended Data Fig. 4a). The "P/E" class is a rotated 70S ribosome with only one hybrid P/E-site tRNA (Extended Data Fig. 7k), and is expected to be an intermediate state during ribosome recycling[93,97-99]. No other 70S class in the recycling phase was detected. The "P/E" state thus may represent one of the rate-limiting steps during ribosome recycling, *i.e.* the next step of EF-G or ribosome recycling factor (RRF) binding to the ribosome in "P/E" state[25]. The "Dim30S-50S" class has a slim 30S subunit and shows no clear tRNA density (Extended Data Fig. 4a, class 13). A class of 70S ribosomes was found to have unclear density near the P site (Extended Data Fig. 4a, class 12). The densities for these two classes were of too low resolution to be unambiguously interpreted. The free 50S could be further classified into two classes: with and without RRF (Extended Data Fig. 4a, classes 50 and 51). For the "50S-RRF" class, the density for the three helices of RRF's domain I is clearly resolved, but domain II is blurred (Extended Data Fig. 7l).

Detailed information for maps resulting from classification and the corresponding models for the four datasets (untreated and cells treated with 3 antibiotics) is provided in Supplementary Tables 3-6.

Supplementary Table 3 | Cryo-EM data collection, refinement and validation statistics of ribosome classes in native untreated cells.

	#1 70S class "P, E" in untreated cells EMD-13272 PDB 7PAH	#2a 70S class "P" in untreated cells EMD-13273 PDB 7PAI	#2e 70S class "EF-Tu •tRNA, P, E" in untreated cells EMD-13274 PDB 7PAJ	#3 70S class "EF-Tu •tRNA, P" in untreated cells EMD-13275 PDB 7PAK	#4 70S class "A, P" in untreated cells EMD-13276 PDB 7PAL	#5 70S class "A*, P/E" in untreated cells EMD-13277 PDB 7PAM	#6a 70S class "A/P, P/E" in untreated cells EMD-13278 PDB 7PAN	#6e 70S class "EF-G, A*, P/E" in untreated cells EMD-13279 PDB 7PAO
**Data collection and processing**								
Magnification	81,000							
Voltage (kV)	300							
Electron exposure (e⁻/Å²)	115 to 135							
Defocus range (μm)	-1.5 to -3.5							
Pixel size (Å)	1.7005							
Symmetry imposed	C1							
Initial particle images (no.)	109,990	109,990	109,990	109,990	109,990	109,990	109,990	109,990
Final particle images (no.)	1,803	6,223	4,634	12,464	32,086	6,587	1,449	3,181
Map resolution (Å) FSC threshold 0.143	9.5	6.7	7.3	5.3	4.7	6.8	9.7	7
Map resolution range (Å)	8 to 13	5.5 to 9	6 to 10	4.5 to 7	4 to 6	5.5 to 9	8 to 14	5 to 12
Map sharpening B factor (Å²)	-100	-79	-87	-22	-33	-73	-100	-36
**Model refinement**								
Initial model used (PDB code)	7OOC, 7OOD, 4V7C, 3J9W	7OOC, 7OOD, 4V7C	7OOC, 7OOD, 4V7C, 4V5L	7OOC, 7OOD, 4V7C, 4V5L	7OOC, 7OOD, 4V7C	7OOC, 7OOD, 4V7C	7OOC, 7OOD, 4V7C	7OOC, 7OOD, 4V7C, 4V7D
Model resolution (Å) FSC threshold = 0.143	10.2	6.5	7.2	5.3	4.7	6.7	10.5	7.3
Model vs. map correlation coefficient (cc_mask)	0.84	0.81	0.83	0.8	0.79	0.83	0.84	0.82
Model composition								
Non-hydrogen atoms	146,172	144,554	150,811	149,193	146,387	146,172	146,172	152,033
Protein residues	5,922	5,922	6,315	6,315	5,927	5,922	5,922	6,673
RNA residues	4,628	4,552	4,704	4,628	4,637	4,628	4,628	4,628
Ligands	0	0	0	0	0	0	0	0
B factors (Å²) (mean)								
Protein	408.35	56.70	109.19	58.06	25.58	95.39	450.67	182.38
RNA	432.62	77.71	131.45	68.42	32.02	116.53	460.27	144.62
R.m.s. deviations								
Bond lengths (Å)	0.005	0.002	0.005	0.002	0.002	0.006	0.002	0.004
Bond angles (°)	0.635	0.566	0.612	0.595	0.544	0.636	0.571	0.563
Validation								
MolProbity score	2.46	2.26	2.36	2.22	2.14	2.31	2.34	2.24
Clashscore	29.32	21.26	23.86	18.10	15.25	23.03	23.83	20.92
Poor rotamers (%)	0.06	0.04	0.04	0.00	0.06	0.06	0.04	0.03
Ramachandran plot								
Favored (%)	91.56	93.16	91.90	92.71	92.91	92.78	92.49	93.47
Allowed (%)	8.37	6.84	8.00	7.28	7.08	7.18	7.49	6.5
Disallowed (%)	0.07	0	0.10	0.02	0.02	0.03	0.02	0.03

Supplementary Table 3 Continued | Cryo-EM data collection, refinement and validation statistics of ribosome classes in native untreated cells.

	#7<br>70S class<br>"EF-G, A/P, P/E"<br>in untreated cells<br>EMD-13280<br>PDB 7PAQ	#8<br>70S class<br>"EF-G, ap/P, pe/E"<br>in untreated cells<br>EMD-13281<br>PDB 7PAR	#11<br>70S class<br>"P/E"<br>in untreated cells<br>EMD-13282<br>PDB 7PAS	#12<br>70S with unknown P-site density"<br>in untreated cells<br>EMD-13283	#13<br>70S class with dim 30S subunit<br>in untreated cells<br>EMD-13284	#50<br>free 50S<br>in untreated cells<br>EMD-13285<br>PDB 7PAT	#51<br>"50S-RRF"<br>in untreated cells<br>EMD-13286<br>PDB 7PAU
**Data collection and processing**							
Magnification	81,000						
Voltage (kV)	300						
Electron exposure ($e^-/Å^2$)	115 to 135						
Defocus range (μm)	-1.5 to 3.5						
Pixel size (Å)	1.7005						
Symmetry imposed	C1						
Initial particle images (no.)	109,990	109,990	109,990	109,990	109,990	109,990	109,990
Final particle images (no.)	1,479	3,324	675	1,484	2,150	15,954	8,203
Map resolution (Å)<br>FSC threshold 0.143	8.9	8.2	16	16	23	9.2	8.3
Map resolution range (Å)	7 to 14	6.5 to 13	15 to 22	14 to 22	18 to 28	6.5 to 12	6 to 12
Map sharpening B factor ($Å^2$)	-70	-83	-200	-200	-200	-200	-67
**Model refinement**							
Initial model used (PDB code)	7OOC, 7OOD, 4V7C, 4V7D	7OOC, 7OOD, 4V7C, 4V7D	7OOC, 7OOD, 4V7C			7OOD	7OOD, 1EH1
Model resolution (Å)<br>FSC threshold = 0.143	9.5	8.7	22.1			10.1	9
Model vs. map correlation coefficient (cc_mask)	0.83	0.84	0.8			0.82	0.84
Model composition							
Non-hydrogen atoms	152,033	152,033	144,492			91,330	92.841
Protein residues	6,673	6,673	5,922			3,467	3.649
RNA residues	4,628	4,628	4,549			2,983	2,983
Ligands	0	0	0			0	0
B factors ($Å^2$) (mean)							
Protein	345.42	189.85	980.09			258.85	194.01
RNA	333.45	191.41	990.86			296.18	204.63
R.m.s. deviations							
Bond lengths (Å)	0.002	0.002	0.005			0.006	0.006
Bond angles (°)	0.587	0.589	0.608			0.546	0.521
Validation							
MolProbity score	2.35	2.36	2.43			2.30	2.28
Clashscore	24.82	24.91	28.83			24.32	22.62
Poor rotamers (%)	0.02	0.02	0.04			0	0
Ramachandran plot							
Favored (%)	92.57	92.39	92.10			93.54	93.42
Allowed (%)	7.4	2.36	7.85			6.46	6.58
Disallowed (%)	0.03	0.06	0.05			0	0

Supplementary Table 4 | Cryo-EM data collection, refinement and validation statistics of ribosome classes in chloramphenicol-treated cells.

	#2a 70S class "P" in Cm-treated cells EMD-13410 PDB 7PH9	#3 70S class "EF-Tu•tRNA, P" in Cm-treated cells EMD-13411 PDB 7PHA	#4 70S class "A, P" in Cm-treated cells EMD-13412 PDB 7PHB	#5 70S class "A*, P/E" in Cm-treated cells EMD-13413 PDB 7PHC	#13 70S class with dim 30S subunit in Cm-treated cells EMD-13414
**Data collection and processing**					
Magnification	81,000				
Voltage (kV)	300				
Electron exposure (e⁻/Å^2)	115 to 135				
Defocus range (μm)	-1.5 to -3.5				
Pixel size (Å)	1.7005				
Symmetry imposed	C1				
Initial particle images (no.)	21,299	21,299	21,299	21,299	21,299
Final particle images (no.)	2,218	1,786	12,915	1,082	986
Map resolution (Å)<br>FSC threshold 0.143	8.7	8.5	4.9	9.9	16.7
Map resolution range (Å)	8 to 12	8 to 12	4.5 to 8	9 to 12	16 to 24
Map sharpening B factor (Å^2)	-57	-65	-20	-200	-200
**Model refinement**					
Initial model used (PDB code)	7OOC, 7OOD, 4V7C	7OOC, 7OOD, 4V7C, 4V5L	7OOC, 7OOD, 4V7C, 3J9W	7OOC, 7OOD, 4V7C	
Model resolution (Å)<br>FSC threshold = 0.143	9.3	9	4.9	10.3	
Model vs. map correlation coefficient (cc_mask)	0.82	0.84	0.76	0.8	
Model composition					
Non-hydrogen atoms	144,563	149,202	146,382	146,181	
Protein residues	5,922	6,315	5,922	5,922	
RNA residues	4,552	4,628	4,637	4,628	
Ligands	0	0	1	0	
B factors (Å^2) (mean)					
Protein	176.53	237.58	22.52	466.99	
RNA	194.04	261.40	29.41	470.16	
R.m.s. deviations					
Bond lengths (Å)	0.005	0.005	0.002	0.002	
Bond angles (°)	0.524	0.576	0.497	0.599	
Validation					
MolProbity score	2.21	2.27	2.10	2.29	
Clashscore	18.54	20.45	15.11	21.14	
Poor rotamers (%)	0.02	0.02	0.02	0	
Ramachandran plot					
Favored (%)	93.09	92.63	93.78	92.49	
Allowed (%)	6.91	7.37	6.22	7.49	
Disallowed (%)	0	0	0	0.02	

Supplementary Table 5 | Cryo-EM data collection, refinement and validation statistics of ribosome classes in spectinomycin-treated cells.

	#2a 70S class "P" in Spc-treated cells EMD-13432 PDB 7PI8	#3 70S class "EF-Tu•tRNA, P" in Spc-treated cells EMD-13433 PDB 7PI9	#6a 70S class "A/P, P/E" in Spc-treated cells EMD-13434 PDB 7PIA	#7 70S class "EF-G, A/P, P/E" in Spc-treated cells EMD-13435 PDB 7PIB	#11 70S class "P/E" in Spc-treated cells EMD-13436 PDB 7PIC	#50 free 50S in Spc-treated cells EMD-13431
**Data collection and processing**						
Magnification	81,000					
Voltage (kV)	300					
Electron exposure (e⁻/Å²)	115 to 135					
Defocus range (μm)	-1.5 to -3.5					
Pixel size (Å)	1.053					
Symmetry imposed	C1					
Initial particle images (no.)	13,418	13,418	13,418	13,418	13,418	13,418
Final particle images (no.)	485	2,239	1,119	8,371	721	483
Map resolution (Å)<br>FSC threshold 0.143	8.9	6.3	13.6	4.7	9.1	9.9
Map resolution range (Å)	7 to 10	5.5 to 8	12 to 16	4 to 6	8 to 12	8.5 to 12
Map sharpening B factor (Å²)	-85	-30	-100	-10	-102	-268
**Model refinement**						
Initial model used (PDB code)	7OOC, 7OOD, 4V7C	7OOC, 7OOD, 4V7C, 4V5L	7OOC, 7OOD, 4V7C	7OOC, 7OOD, 4V7C, 4V7D, 4V56	7OOC, 7OOD, 4V7C	
Model resolution (Å)<br>FSC threshold = 0.143	9.2	6.5	16	4.8	9.4	
Model vs. map correlation coefficient (cc_mask)	0.8	0.77	0.73	0.74	0.81	
Model composition						
Non-hydrogen atoms	144,554	149,193	146,172	152,033	144,554	
Protein residues	5,922	6,315	5,922	6,673	5,922	
RNA residues	4,552	4,628	4,628	4,628	4,552	
Ligands	0	0	0	1	0	
B factors (Å²) (mean)						
Protein	270.87	80.29	879.18	47.49	242.97	
RNA	294.78	99.53	857.64	55.39	249.44	
R.m.s. deviations						
Bond lengths (Å)	0.002	0.005	0.002	0.002	0.005	
Bond angles (°)	0.554	0.553	0.662	0.543	0.534	
Validation						
MolProbity score	2.32	2.22	2.49	2.12	2.26	
Clashscore	24.01	20.09	31.3	15.40	22.02	
Poor rotamers (%)	0	0.02	0.04	0.02	0.02	
Ramachandran plot						
Favored (%)	93.09	93.67	91.36	93.47	93.59	
Allowed (%)	6.89	6.33	8.61	6.53	6.41	
Disallowed (%)	0.02	0	0.03	0	0	

Supplementary Table 6 | Cryo-EM data collection, refinement and validation statistics of ribosome classes in pseudouridimycin-treated cells.

	#2a 70S class "P" in PUM-treated cells EMD-13445 PDB 7PIO	#3 70S class "EF-Tu•tRNA, P" in PUM-treated cells EMD-13446 PDB 7PIP	#4 70S class "A, P" in PUM-treated cells EMD-13447 PDB 7PIQ	#5 70S class "A*, P/E" in PUM-treated cells EMD-13448 PDB 7PIR	#6e 70S class "EF-G, A*, P/E" in PUM-treated cells EMD-13449 PDB 7PIS	#7 70S class "EF-G, A/P, P/E" in PUM-treated cells EMD-13450 PDB 7PIT	#13 70S class with dim 30S subunit in PUM-treated cells EMD-13451	#50 free 50S in PUM-treated cells EMD-13452
**Data collection and processing**								
Magnification	64,000							
Voltage (kV)	300							
Electron exposure (e⁻/Å^2)	115 to 135							
Defocus range (μm)	-1.5 to -3.5							
Pixel size (Å)	1.7005							
Symmetry imposed	C1							
Initial particle images (no.)	23,014	23,014	23,014	23,014	23,014	23,014	23,014	23,014
Final particle images (no.)	1,457	1,128	1,534	940	627	8,730	898	5,671
Map resolution (Å)<br>FSC threshold 0.143	9.5	9.3	9.7	12.1	15	5.7	20.7	8.7
Map resolution range (Å)	8 to 12	8 to 12	8.5 to 12	10 to 14	14 to 16	4.5 to 6.5	18 to 24	8 to 10
Map sharpening<br>B factor (Å^2)	-200	-185	-162	-100	-100	-27	-200	-77
**Model refinement**								
Initial model used<br>(PDB code)	7OOC, 7OOD, 4V7C	7OOC, 7OOD, 4V7C, 4V5L	7OOC, 7OOD, 4V7C	7OOC, 7OOD, 4V7C	7OOC, 7OOD, 4V7C, 4V7D	7OOC, 7OOD, 4V7C, 4V7D		
Model resolution (Å)<br>FSC threshold = 0.143	9.1	8.9				5.6		
Model vs. map correlation coefficient (cc_mask)	0.81	0.71	0.84	0.79	0.77	0.79		
Model composition								
Non-hydrogen atoms	144,541	149,130	146,096	146,120	152,020	152,020		
Protein residues	5,922	6,310	5,915	5,918	6,673	6,673		
RNA residues	4,552	4,628	4,628	4,628	4,628	4,628		
Ligands	0	0	0	0	0	0		
B factors (Å^2) (mean)								
Protein	613.10	886.31	402.22	990.93	999.72	76.06		
RNA	597.87	892.11	412.34	993.45	999.99	84.98		
R.m.s. deviations								
Bond lengths (Å)	0.003	0.003	0.005	0.005	0.003	0.003		
Bond angles (°)	0.688	0.726	0.750	0.79	0.772	0.742		
Validation								
MolProbity score	2.28	2.32	2.38	2.42	2.42	2.29		
Clashscore	20.73	22.77	25.21	27.42	26.92	20.14		
Poor rotamers (%)	0.29	0.31	0.39	0.41	0.52	0.42		
Ramachandran plot								
Favored (%)	92.61	92.39	91.84	91.66	91.41	92.02		
Allowed (%)	7.30	7.48	8.09	8.29	8.48	7.93		
Disallowed (%)	0.09	0.13	0.07	0.05	0.11	0.05		

Translation landscapes in *M. pneumoniae*

We consider the frozen-hydrated cells to constitute a snapshot of the steady state of the non-equilibrium system of a living cell[100]. For translation elongation, the steady state is reflected by the relatively stable occurrence frequencies of the elongation intermediates across 356 untreated *M. pneumoniae* cells (Fig. 3a, e). *M. pneumoniae* cells are known to exhibit slow growth rate and long duplication time (as long as 8 hours)[18,89,101,102], potentially contributing to the observation of relatively low variations between different cells (Fig. 3e). Reported numbers of ribosomes per *M. pneumoniae* cell vary from 80 to 863[18,103,104]. Our work provides an estimation of 300 to 500 ribosomes per cell. Through dividing these number by the cellular volume estimated from tomograms, we calculated the averaged concentration of 70S ribosomes in *M. pneumoniae* to be 7,400 ± 1,600 μm^{-3}. This is considerably lower than the numbers reported in other bacteria, *e.g.* 15,000 μm^{-3} in *Spiroplasma melliferum*[105], and 27,000 - 60,000 μm^{-3} in *E. coli*[106]. Additionally, concentrations of translation factors in *M. pneumoniae* (EF-G, ~10,000 μm^{-3}; EF-Tu, ~40,000 μm^{-3}; total tRNAs, ~14,500 μm^{-3})[107,108] are lower compared to *E. coli* (EF-G, ~75,000 μm^{-3}; EF-Tu, ~500,000 μm^{-3}; total tRNAs, ~500,000 μm^{-3})[109-111]. The low concentrations of ribosomes and translation factors possibly reflect the limited protein biosynthesis capacity of *M. pneumoniae* cells, which has been suggested to lead to their slow growth rate[101,102]. The different intracellular concentrations of ribosomes and translation factors, which constitute the translation machinery of the cell, may contribute to different translation landscapes across bacteria.

Spatial analysis of ribosomes and polysomes

A tomogram in our data can cover 70% to 90% of the volume of a *M. pneumoniae* cell. Overall, ribosomes distributed uniformly throughout the cell (Fig. 4a, Extended Data Fig. 12a), except for their exclusion from the attachment organelle region[112] (Fig. 1a). A nucleoid region present in most model bacterial cells, such as *E. coli,* is suggested to exclude most translating ribosomes[109,113]. This separation, however, was not visible in *M. pneumoniae*, possibly because of its lack of a defined nucleoid[112,114,115].

The functional state determined for each ribosome in *M. pneumonia* can be spatially mapped into the cellular volume (Fig. 4a, Extended Data Fig. 12a). First, we analysed the formation of polysomes by plotting the distribution of relative positions of all neighbouring ribosomes for each ribosome in the tomogram, considering only the ribosome center to center distance (Extended Data Fig. 12b). Although polysomes can be detected and annotated based on center-to-center distance cut-off, it is impossible to obtain the order of ribosomes within the polysome. To address this issue, a polysome detection method based on the distance from mRNA exit of one ribosome to the mRNA entry of following ribosomes was developed (Extended Data Fig. 12c). This method takes the orientations of

ribosomes into consideration and provides the valuable sequential information for the annotated polysome. An additional advantage is that false annotation due to random position between ribosomes in close proximity can be reduced when considering the mRNA exit-to-entry distance as a criterion for polysome annotation. To determine the most appropriate criterion for polysome detection, we calculated the distance distribution for all neighbouring ribosomes and found a peak corresponding to polysomes (Extended Data Fig. 12d). Next, we tested different distance thresholds, ranging from 2 nm to 10 nm, to define polysomes (Extended Data Fig. 12e). The distance criterion of 7 nm provided the best recovery rate and detection accuracy based on visual inspection. The percentage of ribosomes detected as polysomes (26.2%) is consistent with literature reporting that polysomes are often found at relatively low abundance (~30%) in bacteria[116,117]. Most polysomes detected in *M. pneumoniae* cells consist of two to four ribosomes (Fig. 4e), in agreement with a previous work that monitored real-time translation in living cells using fluorescent microscopy[118]. The average distances from the mRNA exit site of the preceding ribosome (i) to the mRNA entry site of the following ribosome (i+1) is 4.2 ± 1.4 nm for "t-t" pairs (9,100 ribosome pairs), and 5.4 ± 1.5 nm for "t-b" pairs (2,491 ribosome pairs). In general, the "t-t" configuration poses tighter packing of ribosomes compared to the "t-b" configuration.

Coordination of translation elongation within the polysome

To probe possible coordination of translation elongation in polysomes, we analysed the frequencies of different state combinations in sequential ribosome pairs in polysomes. The experimental ribosome state pair frequencies show similar global distribution to either the theoretical state pair frequencies calculated from individual state frequencies, or the state pair frequencies of shuffled polysomes (Extended Data Fig. 13a-c). This suggests that on the global level, there is no synchronization of elongation states within polysomes (also see Fig. 4c, d). The shuffled pairs were calculated from the shuffled polysome matrices (Methods, Extended Data Fig. 13d), and represent the distribution of pair frequencies as expected if polysomes assemble randomly. The polysome matrix shuffling procedure allowed us to estimate statistical significance of the differences between the experimental and the shuffled state pair frequencies with a permutation test, where the *p*-value represents the fraction of random permutations in which the state pair frequency was smaller (or larger) than the experimentally observed one (Extended Data Fig. 13d, e, Supplementary Table 7). The results demonstrated that there is a number of pairs for which the experimental value is significantly different from the shuffled value, though most of these differences are between low-frequency pairs. Notably, the majority of these pairs (20 out of 22) include ribosome states that require elongation factor binding to proceed in the elongation cycle (states 1, 2a, 5, 6a). For these four states, the number of pairs in which the ribosome in this state is a preceding ribosome is significantly lower than expected, and the number of pairs in which the ribosome in this state is a following ribosome is significantly higher than expected

(Extended Data Fig. 13e, f). Moreover, we found that this asymmetry in state distribution for two adjacent ribosomes in the polysome becomes more pronounced as the distance between the two ribosomes decrease (Extended Data Fig. 13g). Indeed, for polysomes defined with lower distance thresholds, the ratio of the number of pairs in which the state is a preceding ribosome to the number of pairs in which the state is a following ribosome decreases for states 1, 2a, 5 and 6a, but increases for the other states (Extended Data Fig. 13h). These observations suggested that elongation within polysomes represents a local coordination mechanism that requires close proximity between two neighbouring ribosomes.

Supplementary Table 7 | Permutation *p*(FDR) for fold difference between experimental and shuffled polysome pair frequencies.

Class pairs	1	2a	2e	3	4	5	6a	6e	7	8
**1**	0.0567	0.4355	0.2888	0.1418	0.0140	0.2472	0.2532	0.4355	0.1020	0.3040
**2a**	0.0022	0.0007	0.4576	0.0007	0.0007	0.1078	0.0007	0.0829	0.4277	0.3709
**2e**	0.3709	0.2780	0.0104	0.2780	0.4355	0.0175	0.2472	0.0787	0.0091	0.3709
**3**	0.1111	0.0787	0.0158	0.0007	0.3929	0.0230	0.3491	0.0163	0.2888	0.4481
**4**	0.0018	0.0007	0.0993	0.0359	0.1371	0.0007	0.0007	0.0244	0.2812	0.1371
**5**	0.1300	0.1252	0.0007	0.0007	0.0007	0.0007	0.0207	0.2547	0.0048	0.0007
**6a**	0.4759	0.1278	0.2532	0.0032	0.0007	0.1527	0.0635	0.4355	0.1055	0.0374
**6e**	0.0878	0.1252	0.3946	0.1995	0.2472	0.0133	0.2472	0.0747	0.4500	0.0244
**7**	0.0330	0.3491	0.2871	0.2888	0.4355	0.0035	0.3091	0.3819	0.1798	0.1177
**8**	0.1527	0.4355	0.2547	0.1371	0.4355	0.0018	0.0018	0.2888	0.4701	0.4384

p-values corrected with Benjamini-Hochberg method. FDR, false discovery rate.

Ribosomal protein L9 is conserved across bacteria, but is absent in archaea and eukaryotes[52,119]. L9 has been demonstrated to be flexible, with two RNA binding domains connected by a long helix[51,120,121]. In 70S ribosome crystals, L9 competes with translational GTPases on the following ribosome, preventing crystallization of 70S ribosomes together with these elongation factors[122]. In our tightly packed di-ribosome structures with better resolved ribosome-ribosome interface, the C-terminal domain of L9 is in contact with the 16S rRNA of the following ribosome (Fig. 4h, Extended Data Fig. 12j, k), similar to the packing in crystals. The extended L9 can generate a steric clash with both EF-G and EF-Tu binding to the following ribosome (Extended Data Fig. 12l, m). The overrepresentation of states prior to EF-G binding is more significant than that of states prior to EF-Tu binding. EF-G mediated translocation is the step where the following ribosome (i+1) moves one codon forward along mRNA and is thereby more likely to interact with the extended L9 of the preceding ribosome (i). In all resolved polysome structures, the following ribosome does not appear to block the L1 stalk opening or tRNA disassociation in the preceding ribosome (Fig. 4h, Extended Data Fig. 12j, k).

Effects of antibiotics on the translation machinery

In addition to the specific effect of antibiotics on the occurrence of translation elongation states, we further analysed their influence on the spatial organization of ribosomes. Antibiotic treatment did not induce a visible change to the overall spatial distribution of ribosomes in *M. pneumoniae* cells. However, analysis of polysomes revealed that antibiotics profoundly influenced the organization of ribosomes translating on the same mRNA molecule. The percentages of detected polysomes and the mRNA entry-to-exit distances between adjacent ribosomes within polysomes under the four different cell conditions are summarized in Supplementary Table 8.

Although the exact concentrations of antibiotics in the cells cannot be determined, the drugs were applied at concentrations much higher than the binding saturation concentrations estimated from previous publications (Cm, dissociation constant K_D = 2-6 μM; Spc, reported saturation concentration = 100 μM; PUM, half maximal inhibitory concentration IC_{50} = 0.1 μM)[42,123,124].

In Cm-treated cells, the Cm density in the three minor classes could not be resolved, possibly due to low resolutions of the maps (Extended Data Fig. 9d). No significant class of free 50S was detected, possibly owing to the inhibition of ribosome dissociation mediated by EF-G and RRF by Cm[125]. The percentage of ribosomes detected as polysomes is 20.63%, similar to the number in untreated cells. This is in agreement with the observation that Cm treatment has limited effect on the formation of polysomes[117]. Within polysomes, the ratio of "t-t" to "t-b" pairs increases upon Cm treatment. For "t-t" pairs, the mean distance from the mRNA exit site of the preceding ribosome (i) to the mRNA entry site of the following ribosome (i+1) is approximately 1 nm shorter (about the length of one codon) compared to the untreated cells (Supplementary Table 8). Therefore, polysome organization becomes about one codon tighter upon Cm treatment. This may be related to Cm's specific inhibition of the peptide transfer step, but not translocation (relative mRNA movement). In the presence of Cm, the following ribosome (i+1) within the polysome may still conduct translocation and move one step further along the mRNA[126].

In Spc-treated cells, the map shows the Spc molecule is in close proximity to the amino acid residue K81 (equals to K26 in *E.coli*) on loop 2 of the ribosomal protein S5 (Fig. 3c). This agrees well with reports showing that mutations in the loop 2 regions confer resistance to Spc and affect translation fidelity[127,128]. Whether Spc is bound to ribosomes of the other minor four classes could not be unambiguously determined due to low resolutions. In the presence of Spc, the percentage of polysomes is lower than that in untreated cells (Supplementary Table 8). More polysomes exhibit the "t-b" arrangement, while the mRNA exit to entry distances for "t-t" and "t-b" pairs remain unchanged in comparison to the untreated cells. It is interesting that the two ribosome-specific antibiotics caused

significantly different polysome rearrangements. Spc specifically inhibits 30S dynamics and mRNA translocation[14]. It therefore limits the possibility for polysomes to become more compacted.

PUM is a nucleoside analogue that specifically competes with UTP for the NTP addition site in RNA polymerases[124]. In contrast to the ribosome-specific antibiotics Cm and Spc, the percentage of polysomes in PUM-treated cells is considerably lower (7.73%, Supplementary Table 8). Moreover, the detected polysomes almost all adopt the tight "t-t" configuration with much shorter distances between adjacent ribosomes. We have previously shown that the PUM-stalled RNAP acts as a physical barrier for the leading ribosome and about 60% of all 70S ribosomes in *M. pneumoniae* form a stalled transcription-translation coupling complex (stalled expressome)[2]. Clash between the leading ribosome and the PUM-stalled RNAP arrests the ribosome in the pre-translocational state (Extended Data Fig. 11b), while the following ribosomes translating on the same nascent mRNA can continue elongating until collision with the preceding ribosome. This collision model explains why polysomes in the presence of the RNAP-specific inhibitor are more compacted. Considering the relatively short lifetime of mRNAs (several minutes on average)[129], it is expected that most free mRNAs in *M. pneumoniae* cells are degraded within the drug treatment time of 15-20 minutes. Therefore, we expect most translating ribosomes to be loaded on the nascent mRNAs that are being transcribed by RNAPs. If more ribosomes start translating on nascent mRNAs and collide with preceding ribosomes, most ribosomes would be detected as tightly packed polysomes. However, only 7.73% of 70S ribosomes are detected as closely assembled polysomes in PUM-treated cells. Apart from the 60% that directly collide with RNAP, more than 30% are individual 70S ribosomes. A possible explanation is that collided ribosomes trigger ribosome rescue pathways[130-132]. As PUM does not directly bind to and influence ribosomes, the released ribosomal subunits can again load on the nascent mRNA, start translating and then collide. Such futile loops continue in treated cells until they cannot be sustained. Taken together, these results suggest that both the elongation state distribution and the spatial organization of the translation machinery are completely reshaped upon different antibiotic treatment.

Supplementary Table 8 | Polysome statistics in native untreated and antibiotic-treated cells.

	**untreated**	**Cm-treated**	**Spc-treated**	**PUM-treated**
Polysome percentage*	26.2%	20.63%	12.83%	7.73%
Distance mRNA exit to entry "t-t"	4.2±1.4 (9,100)	3.2±1.5 (1,959)	4.3±1.5 (580)	2.3±0.9 (635)
Distance mRNA exit to entry "t-b"	5.4±1.5 (2,491)	5.3±1.1 (186)	5.1±1.2 (326)	4.9±1.4 (16)

* Ribosomes that are spatially annotated as polysomes out of all 70S ribosomes. The mean and standard deviation for distances from the mRNA exit site of the leading ribosome to the mRNA entry site of the following ribosome for polysome pairs with "t-t" and "t-b" configurations are displayed in nanometers.

Supplementary Video 1 | Visualizing the translation elongation cycle in native untreated cells.

Maps of ribosome classes within the elongation phase are displayed in a sequential manner to illustrate the structural changes during translation elongation. Maps are low-pass filtered to 10 Å.

Supplementary Video 2 | Structural dynamics of translation elongation in native untreated cells.

The atomic models for ribosome intermediates within the elongation phase in untreated cells are displayed sequentially to illustrate the structural dynamics during the translation elongation cycle.

Supplementary references

82. Timsit, Y., Acosta, Z., Allemand, F., Chiaruttini, C. & Springer, M. The role of disordered ribosomal protein extensions in the early steps of eubacterial 50 S ribosomal subunit assembly. *Int J Mol Sci* **10**, 817-834, doi:10.3390/ijms10030817 (2009).
83. Timsit, Y., Sergeant-Perthuis, G. & Bennequin, D. Evolution of ribosomal protein network architectures. *Sci Rep* **11**, 625, doi:10.1038/s41598-020-80194-4 (2021).
84. Klein, D. J., Moore, P. B. & Steitz, T. A. The roles of ribosomal proteins in the structure assembly, and evolution of the large ribosomal subunit. *J Mol Biol* **340**, 141-177, doi:10.1016/j.jmb.2004.03.076 (2004).
85. Lawrence, M. G. *et al.* The extended loops of ribosomal proteins uL4 and uL22 of Escherichia coli contribute to ribosome assembly and protein translation. *Nucleic Acids Res* **44**, 5798-5810, doi:10.1093/nar/gkw493 (2016).
86. Shasmal, M. & Sengupta, J. Structural diversity in bacterial ribosomes: mycobacterial 70S ribosome structure reveals novel features. *PLoS One* **7**, e31742, doi:10.1371/journal.pone.0031742 (2012).
87. Hentschel, J. *et al.* The Complete Structure of the Mycobacterium smegmatis 70S Ribosome. *Cell Rep* **20**, 149-160, doi:10.1016/j.celrep.2017.06.029 (2017).
88. Li, Z. *et al.* Cryo-EM structure of Mycobacterium smegmatis ribosome reveals two unidentified ribosomal proteins close to the functional centers. *Protein Cell* **9**, 384-388, doi:10.1007/s13238-017-0456-9 (2018).
89. Waites, K. B. & Talkington, D. F. Mycoplasma pneumoniae and its role as a human pathogen. *Clin Microbiol Rev* **17**, 697-728, table of contents, doi:10.1128/CMR.17.4.697-728.2004 (2004).
90. Jobe, A., Liu, Z., Gutierrez-Vargas, C. & Frank, J. New Insights into Ribosome Structure and Function. *Cold Spring Harb Perspect Biol* **11**, doi:10.1101/cshperspect.a032615 (2019).
91. Dubochet, J. *et al.* Cryo-electron microscopy of vitrified specimens. *Q Rev Biophys* **21**, 129-228, doi:10.1017/s0033583500004297 (1988).
92. Frank, J. & Gonzalez, R. L., Jr. Structure and dynamics of a processive Brownian motor: the translating ribosome. *Annu Rev Biochem* **79**, 381-412, doi:10.1146/annurev-biochem-060408-173330 (2010).
93. Martin Schmeing, T. & Ramakrishnan, V. What recent ribosome structures have revealed about the mechanism of translation. *Nature* **461**, 1234-1242, doi:10.1038/nature08403 (2009).
94. Selmer, M. *et al.* Structure of the 70S ribosome complexed with mRNA and tRNA. *Science* **313**, 1935-1942, doi:10.1126/science.1131127 (2006).
95. Yusupova, G., Jenner, L., Rees, B., Moras, D. & Yusupov, M. Structural basis for messenger RNA movement on the ribosome. *Nature* **444**, 391-394, doi:10.1038/nature05281 (2006).
96. Loveland, A. B., Demo, G., Grigorieff, N. & Korostelev, A. A. Ensemble cryo-EM elucidates the mechanism of translation fidelity. *Nature* **546**, 113-117, doi:10.1038/nature22397 (2017).

97. Gao, N., Zavialov, A. V., Ehrenberg, M. & Frank, J. Specific interaction between EF-G and RRF and its implication for GTP-dependent ribosome splitting into subunits. *J Mol Biol* **374**, 1345-1358, doi:10.1016/j.jmb.2007.10.021 (2007).
98. Dunkle, J. A. *et al.* Structures of the bacterial ribosome in classical and hybrid states of tRNA binding. *Science* **332**, 981-984, doi:10.1126/science.1202692 (2011).
99. Zhou, D., Tanzawa, T., Lin, J. & Gagnon, M. G. Structural basis for ribosome recycling by RRF and tRNA. *Nat Struct Mol Biol* **27**, 25-32, doi:10.1038/s41594-019-0350-7 (2020).
100. Qian, H. & Beard, D. A. Thermodynamics of stoichiometric biochemical networks in living systems far from equilibrium. *Biophys Chem* **114**, 213-220, doi:10.1016/j.bpc.2004.12.001 (2005).
101. Yus, E. *et al.* Impact of genome reduction on bacterial metabolism and its regulation. *Science* **326**, 1263-1268, doi:10.1126/science.1177263 (2009).
102. Maier, T. *et al.* Quantification of mRNA and protein and integration with protein turnover in a bacterium. *Mol Syst Biol* **7**, 511, doi:10.1038/msb.2011.38 (2011).
103. Seybert, A., Herrmann, R. & Frangakis, A. S. Structural analysis of Mycoplasma pneumoniae by cryo-electron tomography. *Journal of Structural Biology* **156**, 342-354, doi:https://doi.org/10.1016/j.jsb.2006.04.010 (2006).
104. Catrein, I. & Herrmann, R. The proteome of Mycoplasma pneumoniae, a supposedly "simple" cell. *Proteomics* **11**, 3614-3632, doi:10.1002/pmic.201100076 (2011).
105. Ortiz, J. O., Forster, F., Kurner, J., Linaroudis, A. A. & Baumeister, W. Mapping 70S ribosomes in intact cells by cryoelectron tomography and pattern recognition. *J Struct Biol* **156**, 334-341, doi:10.1016/j.jsb.2006.04.014 (2006).
106. Bakshi, S., Siryaporn, A., Goulian, M. & Weisshaar, J. C. Superresolution imaging of ribosomes and RNA polymerase in live Escherichia coli cells. *Mol Microbiol* **85**, 21-38, doi:10.1111/j.1365-2958.2012.08081.x (2012).
107. Wodke, J. A. *et al.* MyMpn: a database for the systems biology model organism Mycoplasma pneumoniae. *Nucleic Acids Res* **43**, D618-623, doi:10.1093/nar/gku1105 (2015).
108. Stülke, J. *MycoWiki database* <http://mycowiki.uni-goettingen.de> (2021).
109. Bakshi, S., Choi, H. & Weisshaar, J. C. The spatial biology of transcription and translation in rapidly growing Escherichia coli. *Front Microbiol* **6**, 636, doi:10.3389/fmicb.2015.00636 (2015).
110. Schmidt, A. *et al.* The quantitative and condition-dependent Escherichia coli proteome. *Nat Biotechnol* **34**, 104-110, doi:10.1038/nbt.3418 (2016).
111. Mustafi, M. & Weisshaar, J. C. Simultaneous Binding of Multiple EF-Tu Copies to Translating Ribosomes in Live Escherichia coli. *mBio* **9**, doi:10.1128/mBio.02143-17 (2018).
112. Krause, D. C. *et al.* Electron cryotomography of Mycoplasma pneumoniae mutants correlates terminal organelle architectural features and function. *Mol Microbiol* **108**, 306-318, doi:10.1111/mmi.13937 (2018).
113. Castellana, M., Hsin-Jung Li, S. & Wingreen, N. S. Spatial organization of bacterial transcription and translation. *Proc Natl Acad Sci U S A* **113**, 9286-9291, doi:10.1073/pnas.1604995113 (2016).

114. Seto, S., Layh-Schmitt, G., Kenri, T. & Miyata, M. Visualization of the attachment organelle and cytadherence proteins of Mycoplasma pneumoniae by immunofluorescence microscopy. *J Bacteriol* **183**, 1621-1630, doi:10.1128/JB.183.5.1621-1630.2001 (2001).
115. Trussart, M. *et al.* Defined chromosome structure in the genome-reduced bacterium Mycoplasma pneumoniae. *Nat Commun* **8**, 14665, doi:10.1038/ncomms14665 (2017).
116. Noll, H. The discovery of polyribosomes. *Bioessays* **30**, 1220-1234, doi:10.1002/bies.20846 (2008).
117. Qin, D. & Fredrick, K. Analysis of polysomes from bacteria. *Methods Enzymol* **530**, 159-172, doi:10.1016/B978-0-12-420037-1.00008-7 (2013).
118. Morisaki, T. *et al.* Real-time quantification of single RNA translation dynamics in living cells. *Science* **352**, 1425-1429, doi:10.1126/science.aaf0899 (2016).
119. Lieberman, K. R. *et al.* The 23 S rRNA environment of ribosomal protein L9 in the 50 S ribosomal subunit. *J Mol Biol* **297**, 1129-1143, doi:10.1006/jmbi.2000.3621 (2000).
120. Hoffman, D. W. *et al.* Crystal structure of prokaryotic ribosomal protein L9: a bi-lobed RNA-binding protein. *The EMBO Journal* **13**, 205-212, doi:10.1002/j.1460-2075.1994.tb06250.x (1994).
121. Fischer, N. *et al.* Structure of the E. coli ribosome-EF-Tu complex at <3 A resolution by Cs-corrected cryo-EM. *Nature* **520**, 567-570, doi:10.1038/nature14275 (2015).
122. Selmer, M., Gao, Y. G., Weixlbaumer, A. & Ramakrishnan, V. Ribosome engineering to promote new crystal forms. *Acta Crystallogr D Biol Crystallogr* **68**, 578-583, doi:10.1107/S0907444912006348 (2012).
123. Bilgin, N., Richter, A. A., Ehrenberg, M., Dahlberg, A. E. & Kurland, C. G. Ribosomal RNA and protein mutants resistant to spectinomycin. *The EMBO Journal* **9**, 735-739, doi:10.1002/j.1460-2075.1990.tb08167.x (1990).
124. Maffioli, S. I. *et al.* Antibacterial Nucleoside-Analog Inhibitor of Bacterial RNA Polymerase. *Cell* **169**, 1240-1248 e1223, doi:10.1016/j.cell.2017.05.042 (2017).
125. Pathak, B. K., Mondal, S. & Barat, C. Inhibition of Escherichia coli ribosome subunit dissociation by chloramphenicol and Blasticidin: a new mode of action of the antibiotics. *Lett Appl Microbiol* **64**, 79-85, doi:10.1111/lam.12686 (2017).
126. Ermolenko, D. N., Cornish, P. V., Ha, T. & Noller, H. F. Antibiotics that bind to the A site of the large ribosomal subunit can induce mRNA translocation. *RNA* **19**, 158-166, doi:10.1261/rna.035964.112 (2013).
127. Ilina, E. N. *et al.* Mutation in ribosomal protein S5 leads to spectinomycin resistance in Neisseria gonorrhoeae. *Front Microbiol* **4**, 186, doi:10.3389/fmicb.2013.00186 (2013).
128. Kamath, D., Gregory, S. T. & O'Connor, M. The Loop 2 Region of Ribosomal Protein uS5 Influences Spectinomycin Sensitivity, Translational Fidelity, and Ribosome Biogenesis. *Antimicrob Agents Chemother* **61**, doi:10.1128/AAC.01186-16 (2017).
129. Hui, M. P., Foley, P. L. & Belasco, J. G. Messenger RNA degradation in bacterial cells. *Annu Rev Genet* **48**, 537-559, doi:10.1146/annurev-genet-120213-092340 (2014).

130. Muller, C., Crowe-McAuliffe, C. & Wilson, D. N. Ribosome Rescue Pathways in Bacteria. *Front Microbiol* **12**, 652980, doi:10.3389/fmicb.2021.652980 (2021).
131. Saito, K. *et al.* Ribosome collisions induce mRNA cleavage and ribosome rescue in bacteria. *Nature*, doi:10.1038/s41586-022-04416-7 (2022).
132. Cerullo, F. *et al.* Bacterial ribosome collision sensing by a MutS DNA repair ATPase paralogue. *Nature*, doi:10.1038/s41586-022-04487-6 (2022).
