# Supplementary material for: Visualizing translation dynamics at atomic detail inside a bacterial cell
Source: Nature. 2022 Sep 28;610(7930):205–11. doi: 10.1038/s41586-022-05255-2 (PMC9534751; doi:10.1038/s41586-022-05255-2)
Supplement: Supplementary file 2 — Reporting Summary [file 41586_2022_5255_MOESM2_ESM.pdf]

nature research

Corresponding author(s): Julia Mahamid

Reporting Summary

Nature Research wishes to improve the reproducibility of the work that we publish. This form provides structure for consistency and transparency in reporting. For further information on Nature Research policies, see Authors & Referees and the Editorial Policy Checklist.

Statistical parameters

When statistical analyses are reported, confirm that the following items are present in the relevant location (e.g. figure legend, table legend, main text, or Methods section).

n/a	Confirmed	
☐	☒	The exact sample size (*n*) for each experimental group/condition, given as a discrete number and unit of measurement
☐	☒	An indication of whether measurements were taken from distinct samples or whether the same sample was measured repeatedly
☐	☒	The statistical test(s) used AND whether they are one- or two-sided<br>*Only common tests should be described solely by name; describe more complex techniques in the Methods section.*
☒	☐	A description of all covariates tested
☐	☒	A description of any assumptions or corrections, such as tests of normality and adjustment for multiple comparisons
☐	☒	A full description of the statistics including central tendency (e.g. means) or other basic estimates (e.g. regression coefficient) AND variation (e.g. standard deviation) or associated estimates of uncertainty (e.g. confidence intervals)
☐	☒	For null hypothesis testing, the test statistic (e.g. *F*, *t*, *r*) with confidence intervals, effect sizes, degrees of freedom and *P* value noted<br>*Give P values as exact values whenever suitable.*
☒	☐	For Bayesian analysis, information on the choice of priors and Markov chain Monte Carlo settings
☐	☒	For hierarchical and complex designs, identification of the appropriate level for tests and full reporting of outcomes
☒	☐	Estimates of effect sizes (e.g. Cohen's *d*, Pearson's *r*), indicating how they were calculated
☐	☒	Clearly defined error bars<br>*State explicitly what error bars represent (e.g. SD, SE, CI)*

Our web collection on statistics for biologists may be useful.

Software and code

Policy information about availability of computer code

Data collection	SerialEM 3.7 or 3.8 (10.1016/j.jsb.2005.07.007)
Data analysis	IMOD 4.9.4, doi: 10.1006/jsbi.1996.0013;<br>Warp 1.0.9, doi: 10.1038/s41592-019-0580-y;<br>MATLAB 2016b & 2019b (https://www.mathworks.com);<br>TOM matlab toolbox release-2008, doi: 10.1016/j.jsb.2004.10.006;<br>RELION 3.0.7 & 3.0.8, doi:10.7554/eLife.42166;<br>M 1.0.9, doi: 10.1038/s41592-020-01054-7;<br>SWISS-MODEL web tool (https://swissmodel.expasy.org/; access time April 2020); use of the online tool only, not of any public data<br>ModeRNA 1.7.1 doi.org/10.1093/nar/gkq1320<br>Coot 0.9, doi: 10.1107/S0907444910007493;<br>Phenix 1.18-3845, doi: 10.1107/S2059798319011471;<br>Chimera 1.13.1, doi: 10.1002/jcc.20084;<br>ChimeraX 1.1.1, doi: 10.1002/pro.3943;<br>IUPred2A tool in Python 3.7.7 doi: 10.1093/nar/gky384;<br>PSIPRED (http://bioinf.cs.ucl.ac.uk/psipred/; https://github.com/fabianegli/JPred-big-batch-submission, access time March/April 2021)<br>MolProbity 4.5 distribution in Phenix 1.18 (DOI: 10.1002/pro.3330)<br>Custom code for bioinformatics analysis of ribosomal protein extensions, and statistical analysis of polysome sequences: GitHub

repository https://github.com/mszimmermann/mycoplasma_ribosome.
Custom MATLAB script for polysome annotation: GitHub repository https://github.com/xueliang4906/polysome_detect.

For manuscripts utilizing custom algorithms or software that are central to the research but not yet described in published literature, software must be made available to editors/reviewers upon request. We strongly encourage code deposition in a community repository (e.g. GitHub). See the Nature Research guidelines for submitting code & software for further information.

Data

Policy information about availability of data

All manuscripts must include a data availability statement. This statement should provide the following information, where applicable:
- Accession codes, unique identifiers, or web links for publicly available datasets
- A list of figures that have associated raw data
- A description of any restrictions on data availability

Cryo-EM maps were deposited in the Electron Microscopy Data Bank (EMDB) under accession numbers: 13234, 13272, 13273, 13274, 13275, 13276, 13277, 13278, 13279, 13280, 13281, 13282, 13283, 13284, 13285, 13286, 13410, 13411, 13412, 13413, 13414, 13431, 13432, 13433, 13434, 13435, 13436, 13445, 13446, 13447, 13448, 13449, 13450, 13451, 13452, 13287, 13288, 13289. Models were deposited in the Protein Data Bank (PDB) under accession numbers: 7OOC, 7OOD, 7P6Z, 7PAH, 7PAI, 7PAJ, 7PAK, 7PAL, 7PAM, 7PAN, 7PAO, 7PAQ, 7PAR, 7PAS, 7PAT, 7PAU, 7PH9, 7PHA, 7PHB, 7PHC, 7PI8, 7PI9, 7PIA, 7PIB, 7PIC, 7PIO, 7PIP, 7PIQ, 7PIR, 7PIS, 7PIT. Maps and atomic models used from previous studies were obtained from EMDB (11998, https://www.emdataresource.org/EMD-11998; 11999, https://www.emdataresource.org/EMD-11999) and PDB (3J9W, https://www.rcsb.org/structure/3j9w; 1DIV, https://www.rcsb.org/structure/1div; 4V63, https://www.rcsb.org/structure/4v63; 1ZAV, https://www.rcsb.org/structure/1zav; 5MMJ, https://www.rcsb.org/structure/5mmj; 4YBB, 1EH1https://www.rcsb.org/structure/4ybb; 4V7C, 1EH1https://www.rcsb.org/structure/4v7c; 4V7D, https://www.rcsb.org/structure/4v7d; 4V5L, https://www.rcsb.org/structure/4v5l; 1EH1, https://www.rcsb.org/structure/1eh1). The Mycoplasma pneumoniae M129 protein and RNA sequences are from NCBI Reference Sequence NC_000912.1(https://www.ncbi.nlm.nih.gov/nuccore/NC_000912.1; access time March 2020).

Field-specific reporting

Please select the best fit for your research. If you are not sure, read the appropriate sections before making your selection.

☒ Life sciences ☐ Behavioural & social sciences ☐ Ecological, evolutionary & environmental sciences

For a reference copy of the document with all sections, see nature.com/authors/policies/ReportingSummary-flat.pdf

Life sciences study design

All studies must disclose on these points even when the disclosure is negative.

Sample size

No sample size calculation was performed. The untreated, chloramphenicol(Cm)-treated and pseudouridimycin(PUM)-treated cellular tomograms were collected in a previous study (DOI: 10.1126/science.abb3758). The spectinomycin-treated data is of a similar size as the other two antibiotic datasets. For each data collection, at least 3 grids were prepared, and typically only 1 of these was used for final data collection. In one grid, there are at least few hundreds of cells that can be used for data collection and only a small percentage were used. The sample size for each experiment was considered sufficient as the obtained maps were resolved at resolutions close to the pixel size (Nyquist) limit of the data. After extensive classification, most classes contain more than one thousand particles, which are expected to ensure reliable classification results in RELION (DOI: 10.1016/bs.mie.2016.04.012). These factors indicate that the current data size is sufficient for the structural and computational analysis presented here.

Data exclusions

For cryo-ET data collection, grids with thick ice or severe mechanistic deformation were discarded. Cells that are clustered in thick ice areas were excluded, because these cells do not contribute sufficiently high-quality data for the structural analysis.
For data processing, tilt-series that contained ice contaminants or more than 4 tilt images with failed tracking were excluded from the final analysis. Such data represented less than 5% of all imaged cells.
For polysome percentage estimation in native untreated cells, the first two processing sessions of 98 tomograms (out of the total 356 untreated tomograms) are not included. Their visual curation with respect to the template matching hits was more stringent than later processed data for the untreated and antibiotic-treated data which may cause some true hits are excluded in these two sessions. To ensure the percentage comparison is valid, the first 98 untreated tomograms was not used. For all other polysome analysis, the total of 356 from untreated cells were used.

Replication

For cryo-ET sample preparation and data collection, at least 3 grids were prepared for each of the experimental consditionds and cryo-ET data were collected on the grid with the best ice thickness in each of the sessions. All computational experiments were repeated to find out the best setups and reproduce the results. For refinement in RELION or M, jobs were repeated to ensure the same resolution can be achieved. The refinement and post-processing (resolution determination) follows the "gold standard" in the cryo-EM/ET field, i.e. the data is randomly split into to half sets and the half sets are refined independently. The reported resolution is based on FSC at 0.143 between densities based on the half sets. For classification, more than 3 parallel jobs were performed to mitigate variations associated with single jobs and to ensure the classification is exhaustive. More follow-up classification jobs were performed to test the convergence of the previous classification. Reproducibility of classification jobs is validated as presented in Extended data figure 5. For polysome annotation and polysome state analysis, the scripts were run for multiple thresholds (presented in Extended data figure 12) before generating the results presented in the manuscript. For all structural modeling, at least 2 rounds of refinements were done, which are usually followed by validation and visual inspection to confirm the model quality.

Randomization

No complete randomization was performed for cryo-ET sample preparation data collection. The selection of grids/cells for data collection was based on ice thickness, position within the film hole, fiducial bead distribution, etc. For cells in regions meeting these quality standards that

	are required for high-quality cryo-ET data, the subsequent processing was performed without considerations of cell shapes or other visible features. For structure refinement in M or RELION, particles were randomly divided into two half datasets by the software. For classification, particles are first randomly divided evenly into classes by RELION. For bioinformatics analysis, structure modeling, and polysome annotation, randomization is not relevant because all were performed according to the confirmed sequences, maps and coordinates. For translation elongation state distribution analysis in polysomes, randomization was performed by reshuffling the polysome sequences 10,000 times with the randperm function in MATLAB 2019b.
Blinding	No blinding was performed as the exact identities of the samples need to be known for the analysis.

Reporting for specific materials, systems and methods

Materials & experimental systems

n/a	Involved in the study
☒	☐ Unique biological materials
☒	☐ Antibodies
☒	☐ Eukaryotic cell lines
☒	☐ Palaeontology
☒	☐ Animals and other organisms
☒	☐ Human research participants

Methods

n/a	Involved in the study
☒	☐ ChIP-seq
☒	☐ Flow cytometry
☒	☐ MRI-based neuroimaging
